# RHOA lactylation at oncogenic hotspots promotes oncogenic activity and protein stabilization

**DOI:** 10.1186/s12943-025-02511-7

**Published:** 2025-11-25

**Authors:** Chenglong Ma, Ruocen Liao, Xingyu Chen, Qianhua Cao, Xinyue Deng, Zhijun Dai, Chenfang Dong

**Affiliations:** 1 Department of Pathology and Pathophysiology, and Department of Surgical Oncology (breast center), Key Laboratory of Cancer Prevention and Intervention, Ministry of Education, The Second Affiliated Hospital, Zhejiang University School of Medicine, Hangzhou, 310058 China; 2 Department of Breast Surgery, First Affiliated Hospital, Zhejiang University School of Medicine, Hangzhou, 310058 China; 3 Department of Respiratory and Critical Care Medicine, Key Laboratory of Respiratory Disease of Ningbo, The First Affiliated Hospital of Ningbo University, 59 Liuting Road, Ningbo, 315010 China; 4 Department of Head and Neck Surgery, Otolaryngology & Head and Neck Center, Cancer Center, Zhejiang Provincial People’s Hospital (Affiliated People’s Hospital), Hangzhou Medical College, Hangzhou, 310014 China

**Keywords:** RHOA, Lactylation, USP9X, PCAF, HDAC3

## Abstract

**Background:**

Aberrant RHOA activation drives tumor progression, yet regulatory mechanisms beyond genetic mutations remain poorly defined. Lactylation, a lactate-derived post-translational modification, links metabolic reprogramming to oncogenesis, but its functional mimicry of genetic mutations is unexplored. This study investigates RHOA lactylation at oncogenic hotspots and its role as an "epi-mutation" system.

**Methods:**

RHOA lactylation were identified by pan-lysine lactylation (Kla) antibody-based mass spectrometry. Site-specific lactylation was achieved using an orthogonal Mb-Pyl Kla-RS/Pyl-tRNA pair to incorporate lactyl-lysine at K118/K162 in recombinant RHOA, validated by immunoblotting and fluorescence. Molecular dynamics simulations (AlphaFold 3, BIOVIA DS) analyzed GTPase activity and hydrogen-bond networks. RHOA activity was assessed via ROCK2-RBD pull-down and GTPase assays. Ubiquitination and protein stability were examined using cycloheximide chase and K48/K63-ubiquitin mutants. In vitro lactylation/delactylation assays with PCAF/HDAC3 defined enzyme specificity. In vitro/in vivo functional studies used migration/invasion assays and xenograft models. Clinical relevance was evaluated in breast cancer tissues and survival databases.

**Results:**

We identify lactylation of RHOA at oncogenic mutation hotspots K118 and K162, mediated by the lactate-PCAF/HDAC3 axis. Mechanistically, K118 lactylation constitutively activates RHOA by impairing intrinsic GTPase activity, whereas K162 lactylation stabilizes RHOA protein by competitively antagonizing protein ubiquitination, with USP9X further enhancing stability through deubiquitination. Functionally, RHOA lactylation promotes tumor cell migration, invasion and metastasis. Clinically, RHOA lactylation is elevated in breast tumors versus adjacent tissues. Notably, targeting lactate production (LDHA inhibitor: sodium oxamate) synergized with RHOA-pathway inhibition (ROCK inhibitor: Y-27632) to suppress tumor progression. By employing a site-specific lactylation system, we further identify that lactylation mimics oncogenic mutations by enhancing both RHOA activity and stability, thus proposing that lactylation at mutation-prone sites represents a reversible "epi-mutation" system that recapitulates genetic mutation effects.

**Conclusions:**

RHOA lactylation at K118 (activation) and K162 (stabilization) orchestrated by the PCAF/HDAC3 enzymatic axis, enables constitutive oncogenic signaling to fuel tumor progression. Crucially, we redefine that lactylation at mutation-prone sites functions as a reversible "epi-mutation" system, where metabolic modification dynamically recapitulates oncogenic mutation effects, challenging the genetic/epigenetic dichotomy in oncology and revealing dual targeting of lactylation and canonical RHOA pathways as a potential therapeutic strategy.

**Supplementary Information:**

The online version contains supplementary material available at 10.1186/s12943-025-02511-7.

## Highlights

• RHOA is lactylated at oncogenic mutation hotspots K118/K162 by PCAF and delactylated by HDAC3

• K118 lactylation impairs GTPase to activate RHOA, whereas K162 blocks ubiquitination to stabilize RHOA

• Lactylation phenocopies oncogenic mutations at these sites, driving tumor invasion and metastasis

• Targeting lactate metabolism sensitizes tumors to RHOA-directed therapies

• Lactylation at mutation-prone residues represents a reversible "epi-mutation" system

**Supplementary Information:**

The online version contains supplementary material available at 10.1186/s12943-025-02511-7.

## Background

The Rho GTPase RHOA is a molecular switch that transitions between an active GTP-bound and an inactive GDP-bound conformation [1, 2]. This molecular switch is regulated by the five most conserved G-boxes (G1-G5) that are nucleotide-binding regions [3]. In response to external stimuli, RHOA specifically recruits effector proteins, including ROCK, MLC, LIMK, and cofilin, to initiate downstream signaling, which is essential for actin reorganization, cell motility, cell migration, and metastasis in various cancer types [1, 4].

Oncogenic RHOA mutations, such as those at G17, Y42, R5, L57, C16, and E40 frequently found in lymphomas and gastric adenocarcinomas, impair intrinsic GTPase activity and promote constitutive activation of downstream effectors [1, 2, 5]. These mutations lock RHOA in its GTP-bound state, leading to sustained signaling that drives tumor progression [1, 2, 4]. However, accumulating clinical evidence reveals that wild-type RHOA (WT-RHOA) is frequently hyperactivated in human cancers [4, 6, 7], with particularly elevated activity observed at invasive tumor fronts and metastatic sites [8], suggesting alternative activation mechanisms beyond genetic mutations.

The Warburg effect, characterized by aerobic glycolysis and lactate accumulation in tumors, links metabolic reprogramming to lactylation [9]. Lactylation has emerged as a critical regulator in human diseases such as Alzheimer's disease, inflamation, and tumor development [9–14]. In this study, we demonstrate that RHOA lactylation at oncogenic mutation hotspots (K118/K162) regulated by PCAF-mediated lactylation and HDAC3-mediated delactylation functionally mimics genetic mutations by constitutively activating RHOA and enhancing its protein stability. Importantly, pharmacological inhibition of lactate production sensitizes lactylated RHOA-driven tumors to mutation-targeting therapies. These findings establish RHOA lactylation as a reversible "epi-mutation" system and propose targeting lactylation or/and canonical RHOA pathways as a precision oncology strategy.

## Methods

### Plasmids and antibodies

Human RHOA gene was amplified from SUM159 cDNA library, and sub-cloned into pLVX-puro. Human KCTD13 and LDHA gene were amplified from MDA-MB-231 cDNA library and subcloned into pLVX-Puro vector. pCMV-3 × FLAG-USP9X(human)-Neo (P56773) and pCMV-USP33(human)−3 × Myc-Neo(P71806) were obtained from MiaoLingBio, China. The DNA fragments of Mb-Pyl Klacr-RS/Pyl-tRNA pair were synthesized by Beijing Tsingke Biotech Co., Ltd. Antibodies against RHOA and MLC2 were purchased from Proteintech (catalog no. 10749–1-AP and 10,906–1-AP). Antibody against FLAG was purchased from Sigma-Aldrich (catalog no. F3165). Antibodies against β-actin, Myc-tag, USP9X and phospho-MLC2 were purchased from ABclonal (catalog no. AC038, A9782, AE070 and AP1503). Antibody against HA-tag was purchased from Abcam (catalog no. ab236632). Pan anti-Kla (PTM-1401RM) antibody was purchased from PTM Bio Inc. Goat Anti-Mouse IgG H&L (Alexa Fluor® 488) waspurchased from Abcam (catalog no. ab150113). Antibodies against PCAF and SIRT2 were purchased from Abways (catalog no. CY8354 and CY5545). Antibodies specifically recognizing lactylation at lysines 118 and 162 were prepared commercially by immunizing rabbits at Hangzhou Kelian Rabbit industry.

### Cell culture

All cells we used in this study were obtained from the American Type Culture Collection (Manassas, VA), where the cell lines were authenticated by STR profiling before distribution. MDA-MB-231, SUM159 and HEK293T cells were grown in Dulbecco's modified Eagle's Medium (DMEM) with 10% FBS. All the cells were cultured and stored according to the instruction from the ATCC. For establishing stable transfectants with overexpression of WT or mutated RHOA, stable clones were selected using 300 ng/mL puromycin for 4 weeks.

### Intracellular expression of site-specific fully lactylated proteins

The eukaryotic orthogonal system utilized chimeric ribozymes from two archaeal ribozymes to increase the efficiency of lactyl-lysine incorporation and used four repeated tRNA sequences to enhance tRNA binding to amber codons. The pcDNA vector plasmid expressing the RHOA protein (specifically mutated to TAG at the K118/K162 site) and the chimeric ribozyme plasmid were transfected into cells at a ratio of 3 to 1. After 12 h, the medium was refreshed and 2 mM lactyl-lysine was added. The transfected cells were cultured for 48 h, and the intracellularly expressed lactylated proteins were confirmed with site-specific antibodies and further analyzed.

### 3D mass spectrometry targeting RHOA Kla

For comprehensive lactylation profiling, HEK293T cells stably expressing Flag-tagged RHOA were processed for mass spectrometry analysis by Shanghai Applied Protein Technology Co., Ltd. (Shanghai, China). Cells were rapidly washed with ice-cold PBS and flash-frozen in liquid nitrogen. Protein extraction was performed using RIPA lysis buffer (50 mM Tris–HCl pH 7.4, 150 mM NaCl, 1% NP-40, 0.5% sodium deoxycholate) supplemented with protease inhibitors (Roche) and de-lactylation inhibitors (20 mM nicotinamide). Protein concentration was determined by BCA assay (Beyotime Biotech) and normalized to 2 mg/mL. For immunoprecipitation, 5 mg of total protein was incubated with anti-Flag M2 magnetic beads (Sigma, M8823) overnight at 4 °C with gentle rotation. Beads were washed six times with high-stringency buffer (50 mM Tris–HCl pH 7.4, 500 mM NaCl, 0.1% SDS), and bound proteins were eluted using 0.1% trifluoroacetic acid. Eluates were reduced with 10 mM DTT (30 min, 56 °C), alkylated with 55 mM iodoacetamide (30 min, dark), and digested with sequencing-grade trypsin (Promega, V5111) at 37 °C for 16 h. Lactylated peptides were enriched using Kla-Pan antibody beads (PTM-1404, PTM Bio) with incubation buffer (50 mM HEPES pH 8.5, 100 mM NaCl). After extensive washing, the resulting peptides were then desalted using C18 ZipTips (Millipore) for LC–MS-MS analysis following the guidelines provided by the manufacturer.

Data processing utilized MaxQuant (v2.4.9.0) against the UniProt human database with the following parameters: precursor mass tolerance 20 ppm (first search)/4.5 ppm (main search), fragment mass tolerance 0.04 Da, fixed carbamidomethyl modification, variable modifications including lysine lactylation (+ 72.02113 Da) and methionine oxidation. FDR was controlled at < 1% using target-decoy approach. Peptide-spectrum matches required minimum length of 7 amino acids and Andromeda score > 40. Lactylation sites were validated by manual inspection of MS2 spectra and retention time consistency. Data were further validated using pFind Studio (v3.1.6) with identical parameters to ensure reproducibility.

### Molecular dynamics simulations

RHOA and mutants (RHOA K118Q, K118R and K118N) were first modeled using AlphaFold 3. All simulations were based on one of these modeled RHOA 3D structures. Molecular dynamics simulations were performed using the standard dynamics cascade protocol in BIOVIA® DS in four steps: (i) minimization; (ii) heating and cooling; (iii) equilibrium run; and (iv) production run for all RHOA and mutants as previously reported [15]. The root mean square deviation (RMSD) of Cα atoms and GTP-binding pocket residues, along with the root mean square fluctuation (RMSF) of Cα atoms, were calculated to evaluate structural stability and flexibility. Hydrogen bond occupancies between GTP and key residues (e.g., K118, K162) were quantified using geometric criteria. Trajectory visualization and analysis were performed using UCSF Chimera and DS Visualizer.

### GTPase assay

Intrinsic GTPase activity for WT and mutant RHOA proteins was measured by the Phosphate Assay Kit (Sigma, #MAK113) to continuously measure phosphate release following the manufacturer's recommended protocol. Briefly, 25 uL of a 1:10 dilution was used to determine the inorganic phosphate concentration, 50 uL of the reagent was added to the sample to be measured, and after 30 min in the dark, the OD 630 was measured in a spectrophotometer. Each 96-well plate contains a calibration curve for the assessment of phosphate concentration.

### RHOA activity assay

Cells were grown to 50–70% confluence for three days, washed with ice-cold PBS, and lysed in Mg^2+^ lysis/wash buffer (Sigma-Aldrich, #20–168), and 500 mg of protein lysate was adjusted to 500 ml with MLB. Active RHOA was co-immunoprecipitated using Flag-M2 agarose, and the ROCK2-RHOA binding domain (RBD) bound to active RHOA was detected by electrophoresis and immunoblotting.

### In vitro lactylation assay

Recombinant GST-PCAF (K311-381BG, Sino Biological) was incubated with Flag-RHOA purified from HEK293T cells in reaction buffer (50 mM HEPES pH7.5, 30 mM KCl, 0.25 mM EDTA, 5 mM MgCl₂, 2 mM DTT) containing 20 mM lactyl-CoA (MedChemExpress) at 37 °C for 1 h. Reactions were quenched with 5 × SDS buffer, denatured at 100 °C for 5 min. Samples were resolved by SDS-PAGE and subjected to immunoblotting using specified antibodies to detect lactylation modifications.

### In vitro delactylation assay

Recombinant GST-HDAC3 (H85-30G, Sino Biological) was incubated with Flag-tagged RHOA K118la/K162la (purified from HEK293T cells) in reaction buffer (50 mM HEPES pH 7.5, 1 mM EDTA, 5 mM MgCl₂, 2 mM DTT) at 37 °C for 1 h. Reactions were terminated by adding 5 × SDS loading buffer, followed by boiling at 100 °C for 5 min. Proteins were resolved by SDS-PAGE, transferred to PVDF membranes, and immunoblotted using the indicated antibodies.

### GTP-sepharose binding assay

To assess GTP-binding activity, HEK-293 T cells overexpressing WT-, lactated, or mutant RHOAs were lysed in ice-cold buffer (50 mM Tris–HCl pH 7.4, 150 mM NaCl, 1 mM EDTA, 1% Triton X-100) containing protease inhibitors (Roche) for 1 h at 4 °C with rotation. Lysates were clarified by centrifugation (21,000 × g, 10 min, 4 °C), and equal protein amounts were incubated with 30 μL GTP-agarose beads (Sigma-Aldrich, G9768) for 4 h at 4 °C with gentle rotation. Beads were washed three times with lysis buffer, and bound proteins were eluted in 4 × SDS loading buffer. GTP-bound RHOA was detected by immunoblotting.

### Immunoprecipitation and western blotting

Cells were lysed in 0.3% Nonidet P40 buffer (50 mM Tris–HCl, 150 mM NaCl, pH 7.5) containing inhibitors (1 ug ml^−1^ of aprotinin, 1 mM phenylmethylsulfonyl fluoride, 1 ug ml^−1^ of pepstatin, 1 ug ml^−1^ of leupeptin, 1 mM Na_3_VO_4_, 30 uM TSA, 1 mM NaF, and 15 mM NAM all at final concentrations). Cell debris was removed by centrifugation at 13,000 rpm for 15 min at 4 °C, and the lysate was incubated with agarose conjugated to the corresponding antibodies for 3 h at 4 °C. Immunoprecipitates were washed three times with 0.3% Nonidet P40 buffer, then boiled and analyzed by Western blotting. For Western blotting, protein concentration was measured using Bradford assay kit (Fude Biological Technology). Adjusted protein samples were mixed with loading buffer and electrophoresed on 12% sodium dodecyl sulfate–polyacrylamide (SDS-PAGE) gels. After electrophoresis, proteins were transferred to polyvinylidene fluoride (PVDF) membranes. The membranes were blocked with 5% fat-free milk for 1 h at room temperature. Blocked membranes were incubated with indicated diluted primary antibody following manufacturer's recommendations and gently shaking at 4 °C overnight. The blots were visualized using ECL assay after incubation with secondary antibody on the next day.

### Colony formation assay

The anchorage-independent growth potential of cells was evaluated using a soft agar colony formation assay, as previously described [16]. Briefly, a double-layer agar system was prepared in 24-well plates, consisting of a base layer (0.7% agar in complete medium) and a top layer (0.35% agar in complete medium containing 5 × 10^3^ cells/well). Cells were carefully resuspended in the top agar layer and plated onto the solidified base layer. Plates were incubated at 37 °C in a 5% CO₂ humidified atmosphere for 21–28 days, with fresh medium added twice weekly. Colonies (> 50 μm in diameter) were visualized under a phase-contrast microscope (Nikon Eclipse Ti) at 40 × magnification and quantified using ImageJ software (NIH). Each condition was tested in triplicate, and experiments were repeated three times independently. For quantification, colonies were stained with 0.005% crystal violet for 1 h prior to counting. The colony formation efficiency was calculated as (number of colonies/number of cells seeded) × 100%.

### Migration and invasion assays

Cell migratory and invasive capacities were evaluated using modified Boyden chamber assays (Corning, 8 μm pore size) as previously described [17]. For migration assays, 5 × 10^4^ cells in serum-free medium were seeded into the upper chamber, while the lower chamber contained complete medium with 10% FBS as chemoattractant. For invasion assays, chambers were pre-coated with Matrigel (BD Biosciences, 1:8 dilution) and incubated for 4 h at 37 °C prior to cell seeding. After 24 h incubation at 37 °C with 5% CO₂, non-migrated/invaded cells were removed from the upper membrane surface using cotton swabs. Migrated/invaded cells on the lower surface were fixed with 4% paraformaldehyde for 15 min, stained with 0.1% crystal violet for 20 min, and quantified by counting five random fields per membrane under an inverted microscope (Nikon Eclipse Ti, 100 × magnification). All experiments included three technical replicates and were repeated independently at least three times. Data were analyzed using GraphPad Prism 9.0, with statistical significance determined by two-tailed Student's t-test (*p* < 0.05 considered significant). Normalization was performed against control groups in each experiment.

### Lung metastasis model

Animal experiments were performed according to procedures approved by the Institutional Animal Care and Use Committee at the Zhejiang University. To evaluate the impact of RHOA lactylation on metastatic potential, female NOD-SCID mice (5–6 weeks old) were randomized into experimental groups (*n* = 6/group) and injected via tail vein with 1 × 10⁶ luciferase-labeled tumor cells expressing either wild-type or mutant RHOA (in 100 μL sterile PBS). Metastatic progression was monitored weekly for 4 weeks using the IVIS Lumina III imaging system (PerkinElmer) following intraperitoneal injection of 150 mg/kg D-luciferin (Gold Biotechnology). At the experimental endpoint, mice were euthanized and lungs were perfused with 4% paraformaldehyde. Tissues were either: 1) embedded in paraffin for sectioning (5 μm thickness) and H&E staining to quantify metastatic nodules, or 2) homogenized for molecular analysis. Histopathological evaluation was performed by two blinded investigators using bright-field microscopy (Nikon Eclipse 80i). Metastatic burden was quantified as both total nodule count and relative luminescent signal intensity. Statistical significance was determined by two-tailed Student's t-test (*p* < 0.05) using GraphPad Prism 9, with data presented as mean ± SEM from three independent experiments.

### Statistical analysis

Results were expressed as mean ± SD or SEM as indicated. Comparisons were made by one-way ANOVA or the two-tailed Student's t-test. Correlations were determined by Pearson's correlation and Spearman's rank correlation test. Survival curves were plotted using the Kaplan–Meier method, and differences were measured by the log-rank test. In all statistical tests, *p* < 0.05 was considered statistically significant.

## Results

### RHOA is lactylated at K118 and K162 sites that are prone to oncogenic mutations

**Fig.1:**
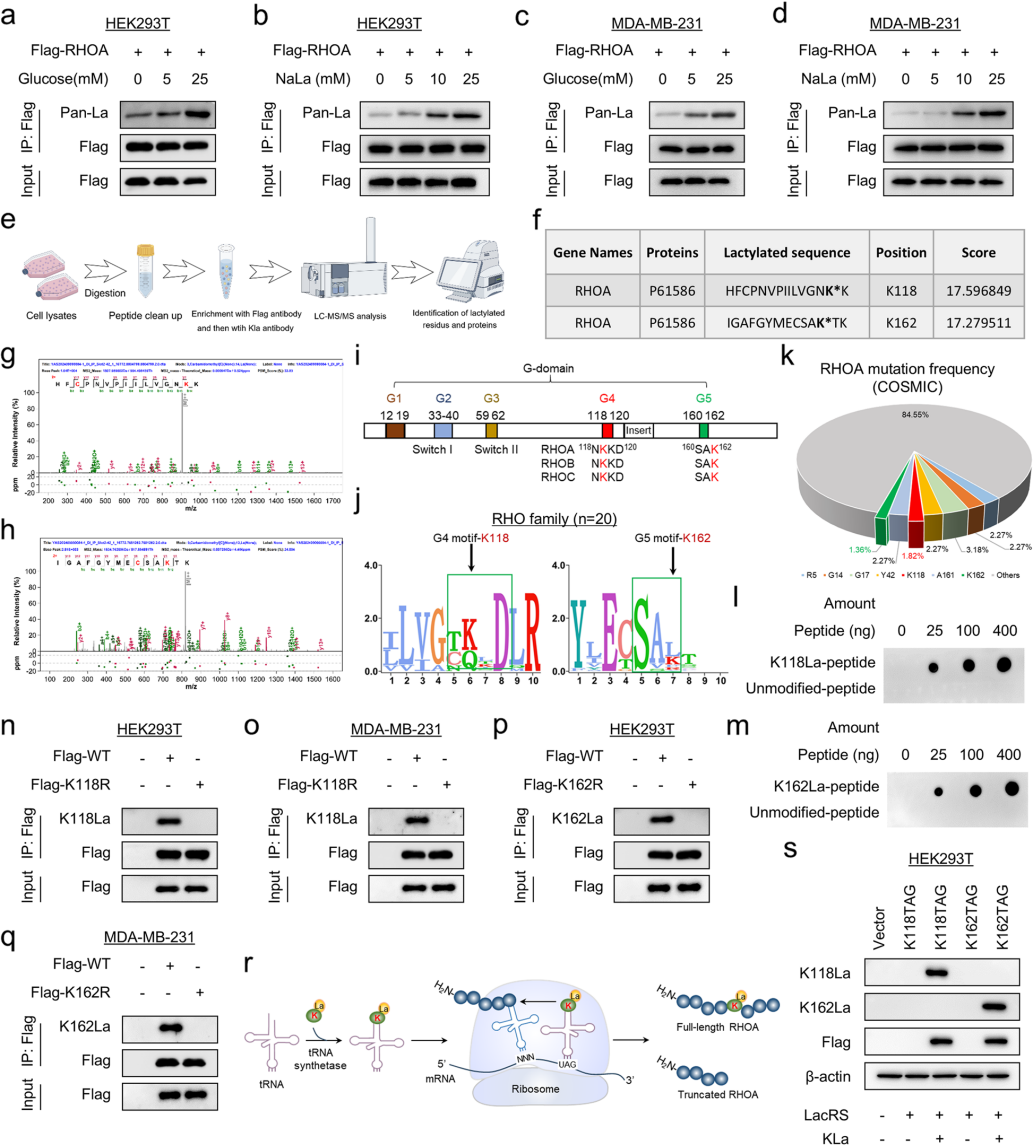
RHOA is lactylated at K118 and K162, sites that are prone to oncogenic mutations. **a**-**d** HEK293T (**a**, **b**) and MDA-MB-231 cells (**c**, **d**) were transfected with Flag-RHOA plasmid and cultured in different concentrations of glucose or sodium lactate for 24 h, followed by immunoprecipitation. The Kla levels of precipitated protein were analyzed by Western blot. **e** Schematic workflow of 3D mass spectrometry experiments. Lysates from HEK293T cells stably expressing RHOA were processed as indicated for 3D mass spectrometry analysis. **f** Table presents the lactylated peptides identified by 3D mass spectrometry. **g**, **h** MS/MS spectra of representative RHOA peptides carrying lactylated K118 (**g**) and K162 (**h**). **i** Schematic representation of the RHOA G domain. Alignment of the G4 and G5 motifs of representative RHOA family members is shown. Conserved lysine residues are highlighted in red. **j** Amino acid sequence logo of conserved G4 and G5 motifs in the RHO family. **k** Frequencies of RHOA mutations in human cancers according to the Catalogue of Somatic Mutations in Cancer (COSMIC). Red and green represent the mutation frequencies of K118 and K162, respectively. **l**, **m** Specificity of K118 (**l**) and K162 (**m**) site-specific lactylation antibodies was examined by dot blotting. **n**-**q** WT-RHOA, K118R or K162R mutants were expressed in HEK293T or MDA-MB-231 cells, and lactylation of K118 (**n**, **o**) and K162 (**p**, **q**) was determined following immunoprecipitation. **r** Schematic diagram describing the generation of site-specific lactylated recombinant RHOA protein using lactyl-lysine tRNA. s Kla was selectively incorporated into RHOA-K118TAG or K162TAG in HEK293T cells using the Mb-Pyl Kla-RS/Pyl-tRNA pair; the expression of lactylated RHOA was analyzed by Western blot

Aberrant activation of RHOA is associated with tumor progression [1, 4]. Our analysis showed that aberrant high expression of RHOA was tightly correlated with poor survival (Supplementary Fig. 1a-c). The relationship between lactate-derived lactylation and tumors has been widely reported [18]. To test whether RHOA can be lactylated, we treated several cell lines with various concentrations of glucose and lactate, showing that RHOA lactylation levels were induced by glucose and lactate in a dose-dependent manner (Fig. 1a-d and Supplementary Fig. 1d-g). To identify the potential RHOA lactylation sites, we used a specific pan-lysine lactylation (Kla) antibody to perform a mass spectrometry (MS)-based screen on lysates of HEK293T cells stably expressing RHOA (Fig. 1e). Two RHOA-derived peptides were identified, which contained lactylation at lysine (K) residues K118 and K162 (Fig. 1f-h). It is worth noting that K118 and K162, located in the G4 box and G5 box, respectively, are major sites in the active regions of the RHOA family (Fig. 1i). Both K118 and K162 are highly conserved in the RHOA family and even the RAS superfamily (Fig. 1j and Supplementary Fig. 1 h). Interestingly, COSMIC (Catalogue of Somatic Mutations in Cancer) analysis revealed that K118 and K162 are two cancer-associated and naturally occurring missense mutation hotspots in RHOA (Fig. 1k). To specifically recognize RHOA lactylation, we generated lactylation-specific antibodies against K118 and K162, respectively (designated as "K118la and K162la"). Dot blot assays showed that both antibodies preferentially recognized the lactylated, but not the unmodified, peptide at their respective sites (Fig. 1l, m). In addition, we mutated K118 and K162 to arginine (R) respectively, showing that both mutations resulted in a dramatic reduction in lactylation (Fig. 1n-q and Supplementary Fig. 1i, j). Together, these findings suggest that RHOA is lactylated at K118 and K162, two major sites that are frequently mutated oncogenically. To unambiguously clarify the effect of lactylation on RHOA, we used a site-specific lactylation system of genetic code expansion to incorporate lactyl-lysine into recombinant RHOA to generate site-specific fully lactylated proteins in vitro (Fig. 1r). We tested the reliability of this system. Specifically, HEK293T cells were transiently co-transfected with genes encoding Flag-RHOA or C-terminally EGFP-tagged RHOA harboring an amber mutation at the permissive sites (RHOA K118TAG or K162TAG) and the PylRS/PylT pair and then incubated in the presence or absence of lactyl-lysine, showing that immunoblot bands and strong fluorescence were only observed in the presence of lactyl-lysine, confirming the site-specific insertion of lactyl-lysine into RHOA (Fig. 1s and Supplementary Fig. 1 k, l).

### K118 lactylation promotes RHOA activity by impairing intrinsic GTPase activity

**Fig.2:**
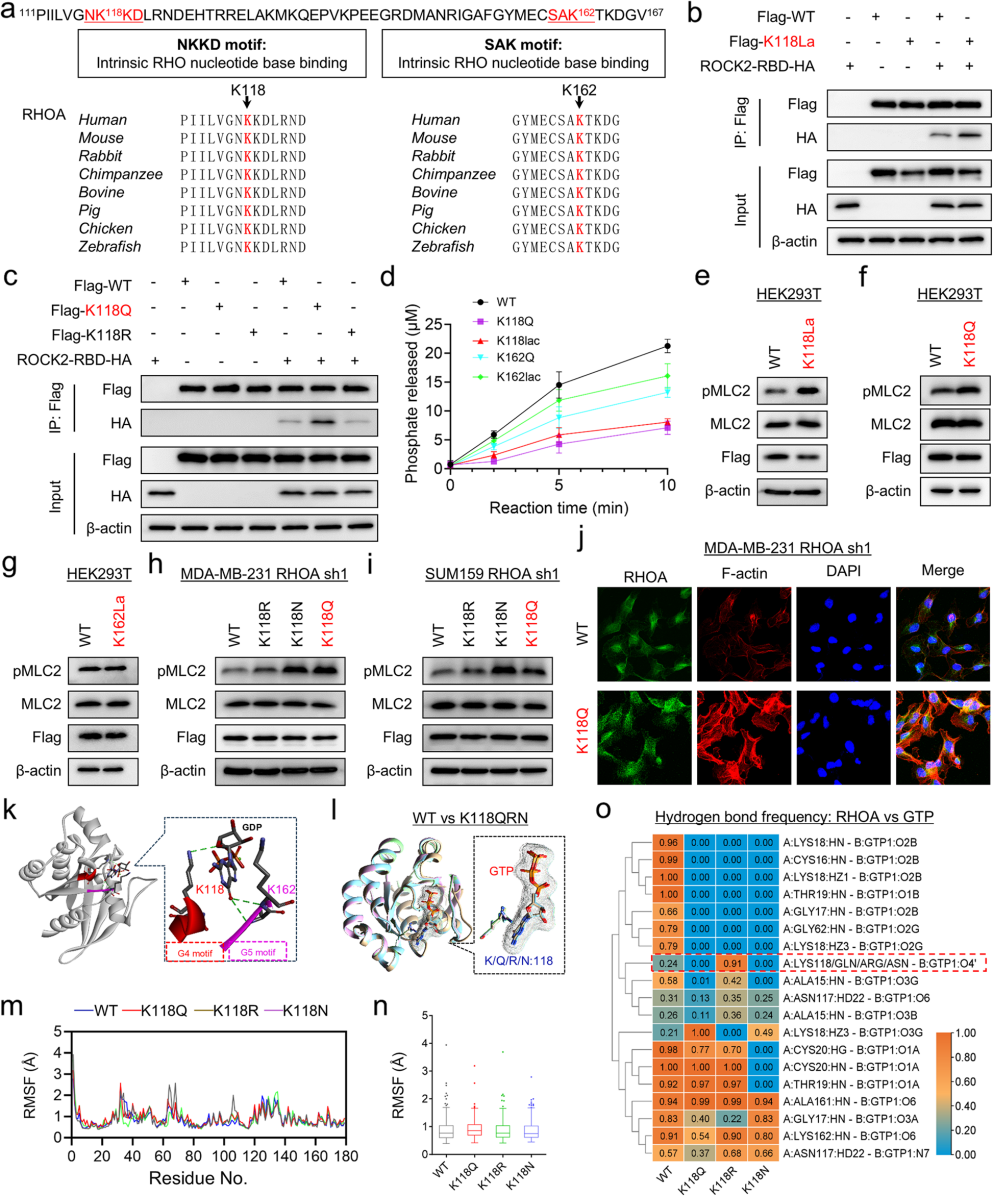
K118 lactylation contributes to accumulation of the active GTP-bound RHOA. **a** Alignment of the lactylation site and adjacent protein sequences of RHOA in different species. **b**, **c** ROCK2-RBD-HA was co-expressed with WT (WT), lactylated (**b**), or different mutant RHOA (**c**) in HEK293T cells, and the immunoprecipitated ROCK2-RBD-HA was examined by Western blotting. **d** WT, lactylated, and mutated RHOA proteins were loaded with GTP, and the rate of intrinsic GTP hydrolysis was analyzed by continuous measurement of phosphate using a colorimetric assay. The concentration of released phosphate was plotted versus time. **e**–**g** Expression of p-MLC2 and MLC2 in HEK293T with WT-RHOA, lactylated RHOA (**e**), K118Q (**f**), or K162Q mutant (**g**) was examined by Western blotting. **h**, **i** Expression of p-MLC2 and MLC2 in shRHOA-expressing MDA-MB231 (**h**) and SUM159 cells (**i**) with WT, lactylated or different mutated RHOA expression was examined by Western blotting. **j** F-actin was stained with Rhodamine Phalloidin (Red) in shRHOA-expressing MDA-MB231 cells with stable WT-RHOA or K118Q mutant expression. **k** Interactions of K118 within the G4 motif and K162 within the G5 motif with GDP-bound RHOA (PDB: 1FTN). Hydrogen bond interactions are shown as dashed lines. **l** Overlay of WT-RHOA (tan), K118Q (sky blue), K118R (pink), and K118N mutants (light green) after modeling. A magnified overlay of GTP structure was shown on the right. **m**,** n** Overlay (**m**) or histogram (**n**) for protein backbone Cα RMSF plots of MD trajectories in WT-RHOA, K118Q, K118R, and K118N mutants. **o** Heatmap for hydrogen-bonding interactions of GTP with WT-RHOA, K118Q, K118R, and K118N mutants. Occupancy is defined as the ratio of the simulation time that a particular hydrogen bond is present

K118 and K162 of RHOA are highly evolutionarily conserved from zebrafish to humans (Fig. 2a). To investigate the effects of both site lactylation and mutations on RHOA activity, we fused the RHOA-binding domain (RBD) of human ROCK2 (amino acids 979 to 1047) that preferentially binds to GTP-loaded RHOA, to HA-tag and glutathione S-transferase (GST), and then tested the effects of lactylation and mutations on binding ability by co-immunoprecipitation and pull-down assays. We found that K118 but not K162 lactylation of RHOA produced by a site-specific lactylation system significantly enhanced the binding ability of RHOA to RBD (Fig. 2b and Supplementary Fig. 2a). Consistently, all K118 but not K162 mutants also resulted in increased binding of RHOA to the RBD (Fig. 2c and Supplementary Fig. 2b-d). RHOA has an intrinsic GTPase activity, and its inactivation activates its downstream signaling. To clarify the effects of K118 lactylation and oncogenic mutations on intrinsic GTP hydrolysis, we measured phosphate release by a phosphate assay kit. Our data showed that the intrinsic hydrolysis capacity of K118 lactylation was comparable to that of the corresponding K118Q, but significantly lower than that of WT-RHOA, K162 lactylation and K162Q (Fig. 2d). We further analyzed the effects of RHOA lactylation and tumor-related mutations on the downstream signaling pathways of HEK293T and breast cancer cell lines. Notably, compared with WT-RHOA and K162 lactylation, K118 lactylation and mutations significantly increased MLC2 phosphorylation (Fig. 2e-i, and Supplementary Fig. 2e, f). RHOA promotes the formation of actin stress fibers, which are critical for cell morphology and adhesion [19]. To determine whether RHOA mutation-mediated ROCK activation affects the cytoskeleton, we stained actin stress fibers. The formation of actin stress fibers was significantly increased in K118Q compared with WT-RHOA (Fig. 2j, and Supplementary Fig. 2 g, h). The side chain ends of lysine acetylation, lysine lactylation and glutamine are composed of a carbonyl group (-C = O) and a nonpolar methyl group (-CH3), a carbonyl group (-C = O) and a polar hydroxyethyl group (-CH3CHOH), and a carbonyl group (-C = O) and a polar amino group (-NH2), respectively (Supplementary Fig. 2i). Based on the polar characteristics of the side chain groups of both lysine lactylation (but not acetylation) and Q, substitution of K with Q may be a better choice to mimic lysine lactylation than acetylation. Indeed, our data showed that the functional trends of lysine lactylation simulated by Q replacing K were consistent with those of the lactylated proteins generated by the site-specific lactylation system (Fig. 2b-j). As the site-specific lactylation system is a transient expression system, for subsequent experiments with a longer duration, we mimicked lysine lactylation using Q to substitute K.

Structural analysis showed that the side chain amino group of K118 in RHOA formed a hydrogen bond with the ribose of guanine nucleotide (Fig. 2k and Supplementary Fig. 3a, b). We used AlphaFold 3 to model RHOA and its mutants (RHOA K118Q, K118R, and K118N). Superposition of the WT-RHOA crystal structure (PDB: 1FTN) and modeled WT-RHOA showed extreme agreement (Supplementary Fig. 3c), supporting the reliability of the modeling. The compilation of RHOA crystal structures showed that WT-RHOA, RHOA lactylation-mimicking variant (K118Q) and tumor-associated mutant variants (K118N and K118R) had extremely similar conformations in GTP and its binding region (Fig. 2l and Supplementary Fig. 3 d). To gain insight into the potential mechanism for promoting RHOA activity, molecular dynamics (MD) simulations were performed to explore the effects of K118 lactylation or mutation on the RHOA structure. RMSF analysis is to understand the flexibility and fluctuation of each residue in the protein structure. Notably, the RMSF values ​​of all K118 mutants were comparable to WT-RHOA (Fig. 2m, n and Supplementary Fig. 3e-g). Indeed, in vitro GTP binding assays showed that the GTP binding capacity of RHOA lactylation (K118La) and mutant (K118Q) did not change significantly compared with WT-RHOA (Supplementary Fig. 3 h). These findings indicate that K118 lactylation or mutations have no significant effect on the binding flexibility and fluctuation.

Hydrogen bonds play an important role in the specificity and directionality of molecular recognition between proteins and their ligands. In RHOA, GTP forms hydrogen bonds with C16, G17, K18, T19, and C20 in the P-loop (G1) of RHOA, G62 in the G3 region, N117 and K118 in the G4 box, and A161 and K162 in the G5 box, respectively [3, 20]. K118 is positively charged prior to lactylation, and its lactylation neutralizes the charge of the residue, disrupting its favorable interaction with GTP. Thus, the effect of K118 lactylation on RHOA may be due to the disruption of electrostatic interactions. As expected, compared with WT-RHOA, the lactylation-mimicking mutation K118Q and the tumor-associated mutation K118N completely lost hydrogen bonding with GTP, whereas the positively charged K118R increased the frequency of hydrogen bonding with GTP (Fig. 2o and Supplementary Fig. 3i). In addition to the hydrogen bonding between residue 118 and GTP, these K118 mutations also eliminated or remarkably reduced the hydrogen bonding occupancy between GTP and residues such as C16, G17, K18, T19, and G62, which are closely related to intrinsic RHOA-GTPase activity (Fig. 2o and Supplementary Fig. 3i). These data indicate that lactylation or mutation of RHOA-K118 results in attenuation or loss of its intrinsic GTPase activity, ultimately leading to RHOA activation.

### K162 lactylation stabilizes RHOA by competitively antagonizing protein ubiquitination

**Fig.3:**
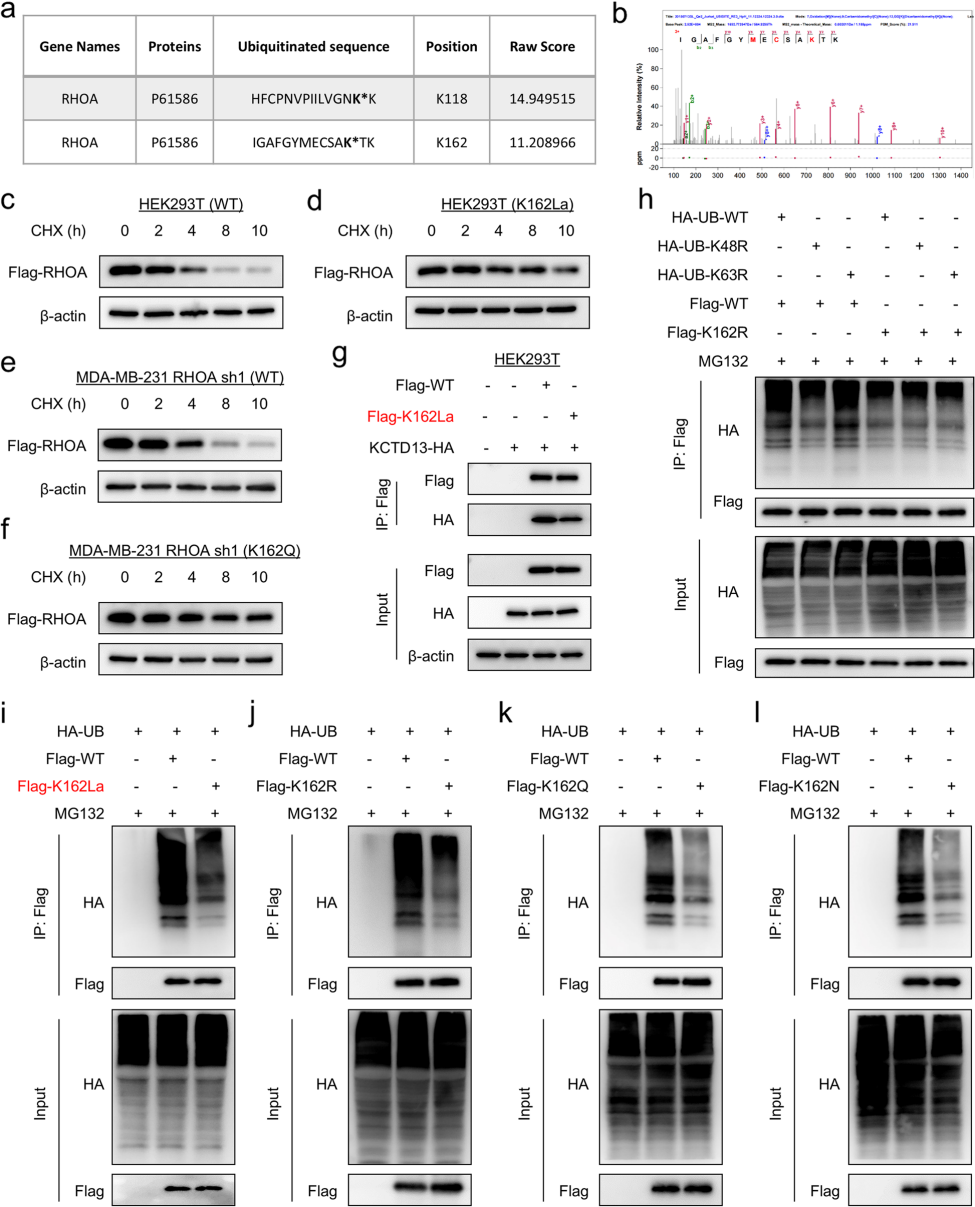
K162 lactylation stabilizes RHOA by competitively antagonizing protein ubiquitination. **a** Table showing the ubiquitinated peptides of RHOA identified by mass spectrometry. **b** MS/MS spectra of representative RHOA peptides carrying K162 ubiquitination. **c**, **d** For cycloheximide (CHX) assay, WT (**c**) or lactylated RHOA (**d**) was expressed in HEK293T cells for 48 h, and then the cells were treated with 25 μg/mL CHX for the indicated time; the expression level of RHOA was examined by Western blotting. **e**, **f** For CHX assay, shRHOA-expressing MDA-MB-231 cells with stable WT-RHOA (**e**) or K162Q mutant expression (**f**) were treated with 25 μg/mL CHX for the indicated time, and then the expression level of RHOA was analyzed by Western blotting. **g** The lysates of HEK293T cells with WT-RHOA, K162La, or/and KCTD13 expression were subjected to immunoprecipitation with Flag-M2 agarose, and then their interactions were analyzed by Western blotting. **h** HEK293T cells were cotransfected with the indicated plasmids, and K48- and K63-linked ubiquitination was measured by ubiquitination assay. **i**-**l** HEK293T cells were cotransfected with the indicated plasmids, ubiquitination of lactylated RHOA (**i**), K162R (**j**), K162Q (**k**), and K162N mutant (**l**) was measured by ubiquitination assay

Mass spectrometry analysis showed that RHOA might be an ubiquitinated protein, and importantly, K118 and K162 were potential ubiquitination sites (Fig. 3a, b and Supplementary Fig. 4a). Indeed, RHOA protein was also significantly increased in cells treated with the proteasome inhibitor MG132, suggesting that RHOA stability is regulated through the ubiquitin–proteasome pathway (Supplementary Fig. 4b). Cycloheximide (CHX) assays also showed that RHOA was an unstable protein (Fig. 3c-f and Supplementary Fig. 4c). However, RHOA K162 lactylation or RHOA K162Q, but not RHOA K118 lactylation or RHOA K118Q, significantly enhanced its protein stability (Fig. 3c-f and Supplementary Fig. 4c-e). BTB/POZ domain-containing protein (KCTD13) is a substrate-specific adapter of a BCR (BTB-CUL3-RBX1) E3 ubiquitin-protein ligase complex capable of binding to RHOA [21]. Interestingly, our analysis showed that abnormally high expression of KCTD13 was negatively correlated with aggressive subtype, drug insensitivity, and poor survival (Supplementary Fig. 4f-j). Immunoprecipitation experiments further confirmed that WT-RHOA could bind to KCTD13, whereas K162La and K162R significantly reduced this binding (Fig. 3g and Supplementary Fig. 4 k). K48-linked ubiquitin chains target proteins for proteasomal degradation, whereas non-K48-linked ubiquitin chains, such as K63-linked polyubiquitin chains, mainly regulate protein activity, localization and other functions [22, 23]. We demonstrated that the K48R ubiquitin mutant, but not K63R, significantly reduced RHOA ubiquitination levels (Fig. 3h), suggesting that K162 ubiquitination is primarily involved in proteasome-mediated protein degradation. To determine the relationship of K162 lactylation or mutations with RHOA protein stability, we performed ubiquitination analysis. The data showed that compared with WT-RHOA, K162 lactylation, K162Q, K162N and K162R ubiquitination levels were significantly reduced (Fig. 3i-l), suggesting that RHOA lactylation and mutations competitively block ubiquitination at this site.

### USP9X targets RHOA for deubiquitination

**Fig.4:**
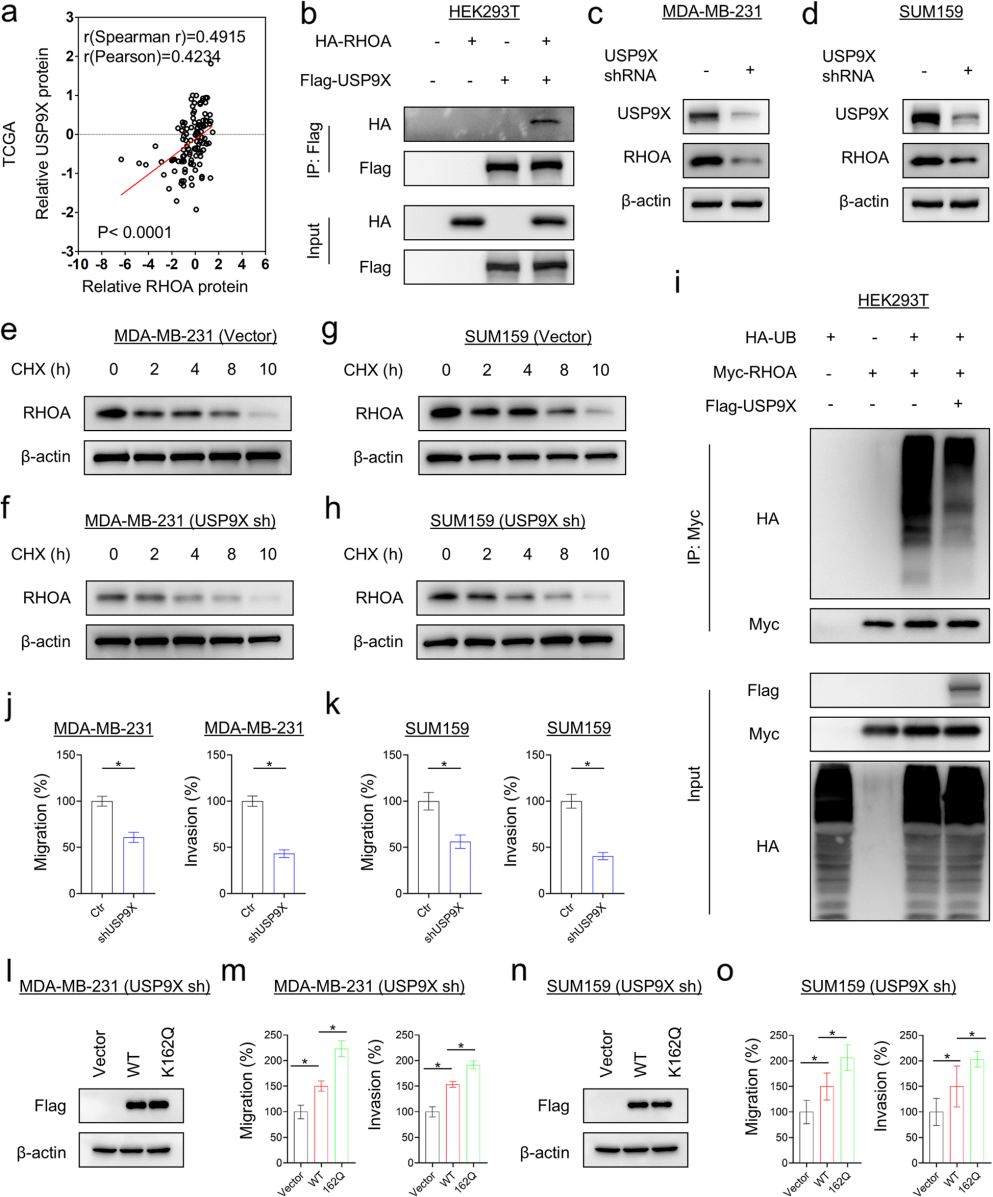
USP9X-mediated RHOA deubiquitylation. **a** Analysis of the TCGA dataset for the expression of RHOA and USP9X. The relative protein level of RHOA was plotted against that of USP9X. Correlations were analyzed using Pearson’s correlation method and Spearman’s rank correlation test. **b** The lysates of HEK293T cells with WT-RHOA or/and USP9X expression were subjected to immunoprecipitation with Flag-M2 agarose, and then their interactions were analyzed by Western blotting. **c**, **d** Expression of RHOA and USP9X was analyzed by Western blotting in MDA-MB-231 (**c**) and SUM159 cells (**d**) with stable empty vector or knockdown of USP9X expression.**e**–**h** For CHX assay, MDA-MB-231 (**e**, **f**) and SUM159 cells (**g**, **h**) with stable empty vector or knockdown of USP9X expression were treated with 25 μg/mL CHX for the indicated time, and then the expression level of RHOA was analyzed by Western blotting. **i** HEK293T cells were co-transfected with the indicated plasmids, ubiquitination of RHOA was measured by ubiquitination assay. **j**, **k** Migratory ability and invasiveness of MDA-MB231 (**j**) and SUM159 cells (**k**) with stable empty vector or knockdown of USP9X expression were analyzed. The percentage of migratory and invasive cells was shown in the bar graph (mean ± SD in three separate experiments). *p < 0.05 by Student’s t-test. **l**, **n** Expression of RHOA and K162Q mutant was analyzed by Western blotting in MDA-MB-231 (**l**) and SUM159 cells (**n**) with stable knockdown of USP9X expression. **m**, **o** Migratory ability and invasiveness of shUSP9X-expressing MDA-MB231 (**m**) and SUM159 cells (**o**) with stable empty vector, WT-RHOA or K162Q mutant were analyzed. The percentage of migratory and invasive cells was shown in the bar graph (mean ± SD in three separate experiments). *p < 0.05 by Student’s t-test

Ubiquitin carboxyl-terminal hydrolase 9X (USP9X) is a deubiquitinase involved both in the processing of ubiquitin precursors and of ubiquitinated proteins [24]. Analysis of protein–protein interaction databases showed that USP9X was a putative RHOA-interacting deubiquitinase (Supplementary Fig. 5a). Clinical data analysis showed that abnormally high expression of USP9X was closely associated with high tumor grade and poor survival rate (Supplementary Fig. 5b-e), and USP9X was positively correlated with RHOA protein expression levels (Fig. 4a). To determine whether USP9X is a potential deubiquitinase that interacts with RHOA, we performed immunoprecipitation, confirming the interaction between USP9X, but not USP33, and RHOA (Fig. 4b and Supplementary Fig. 5f). We further knocked down USP9X expression to examine its effect on RHOA, showing that knockdown of USP9X expression significantly decreased RHOA protein levels (Fig. 4c, d). Consistently, knockdown of USP9X expression significantly reduced its protein stability as shown by CHX experiments (Fig. 4e-h), whereas USP9X expression could partially restore RHOA protein levels as shown by ubiquitination assays (Fig. 4i). We also evaluated the function of USP9X in tumor cells and found that knockdown of USP9X expression significantly inhibited the migration and invasion of MDA-MB231 and SUM159 cells in vitro (Fig. 4j, k). Importantly, expression of the K162Q mutant in USP9X knockdown cell lines more significantly restored these migration/invasion abilities compared to expression of WT-RHOA (Fig. 4l-o). Together, these findings indicate that USP9X is a RHOA deubiquitinase.

### PCAF lactylates, whereas HDAC3 delactylates RHOA

**Fig.5:**
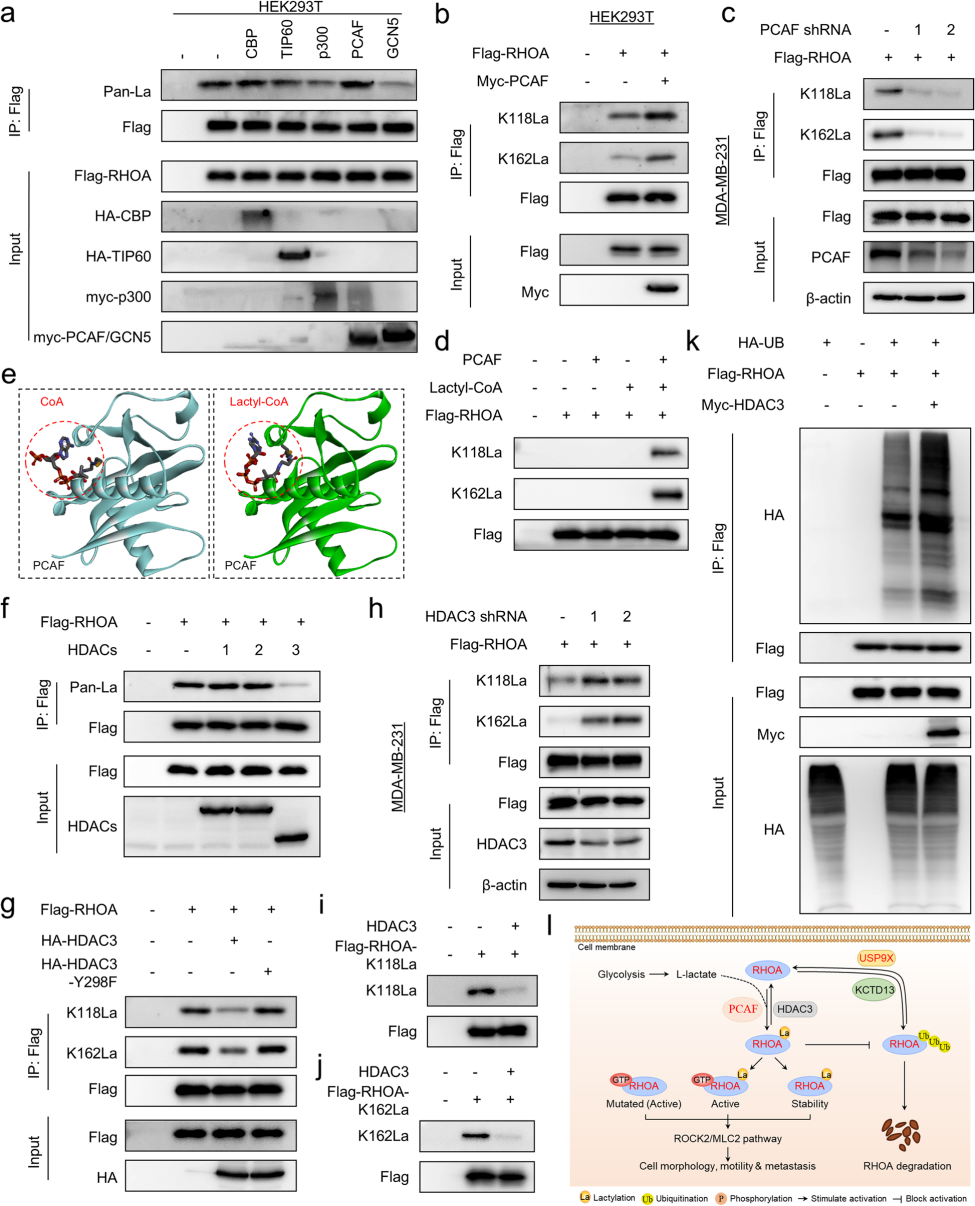
PCAF lactylates, whereas HDAC3 delactylates RHOA. **a** HEK293T cells were co-transfected as indicated, and the lactylation level of RHOA was measured using a pan-Kla antibody. **b** HEK293T cells were transfected with RHOA and/or PCAF plasmids, and the lactylation level of RHOA was measured using site-specific lactylation antibodies. **c** MDA-MB231 cells were co-expressed with RHOA and/or shPCAF. Following immunoprecipitation, the lactylation level of RHOA was measured using a site-specific lactylation antibody. **d** In vitro lactylation assay showing the lysine lactyltransferase activity of PCAF on RHOA-K118 and RHOA-K162. **e** The cofactor pocket of PCAF (PDB: 1CM0) bound to CoA (left) and lactyl-CoA (right). PCAF is shown in cartoon form. The transfer groups in CoA or lactyl-CoA are indicated by red circles**. f** HEK293T cells were co-expressed with RHOA, and indicated HDAC, and the lactylation level of RHOA was measured using a pan-Kla antibody. **g** HEK293T cells were co-expressed with RHOA, and HDAC3 or HDAC3-Y298F (enzymatically inactive), and the lactylation level of RHOA was measured using site-specific lactylation antibodies. **h** MDA-MB231 cells were expressed with RHOA and/or shHDAC3. Following immunoprecipitation, the lactylation level of RHOA was measured using a site-specific lactylation antibody. **i**, **j** In vitro delactylation assay showing the delactylase activity of HDAC3 on RHOA-K118La (**i**) and RHOA-K162La (**j**). **k** HEK293T cells were co-transfected with the indicated plasmids, ubiquitination of RHOA were measured. **l** A proposed model to illustrate the lactylation-mediated regulation of RHOA activity and protein stability (see Discussion)

To screen for lactyltransferases responsible for RHOA lactylation, we experessed five acyltransferases, cAMP response element binding protein (CREB) binding protein (CBP), TIP60 (KAT5), p300 (E1A binding protein, 300 kDa), PCAF (KAT2B), and GCN5 (KAT2A), in HEK293T cells, showing that ectopic expression of PCAF significantly increased RHOA lactylation, whereas other acyltransferases had little effect (Fig. 5a). Notably, PCAF expression significantly promoted, whereas knockdown of PCAF expression decreased the lactation levels of both K118 and K162 of RHOA. (Fig. 5b, c). In vitro lysine lactylation confirmed that PCAF efficiently lactylated wild-type RHOA-K118/162 but shows no activity toward K-to-R/Q/N mutants, exhibiting strict enzymatic specificity (Fig. 5d and Supplementary Fig. 6a, b). These data suggest that PCAF is a potential lysine lactyltransferase responsible for RHOA lactylation. To explore this possibility, we also docked lactyl-CoA into the structure of PCAF (PDB: 1CM0). Lactyl-CoA was well accommodated in the cofactor pocket of PCAF, similar to the structure of the coenzyme A (CoA)-PCAF complex (Fig. 5e). Aminoacyl-tRNA synthetase 1 (AARS1), an enzyme that catalyzes the conjugation of alanine to tRNA (Ala), has been reported to catalyzes the formation of lactate-AMP, which is then transferred to a lysine acceptor residue [13]. Our data showed that AARS1 didn’t affected the lactylation level of RHOA (Supplementary Fig. 6c), suggesting that lactylation of different proteins may be regulated by different acyltransferases.

To identify the RHOA delactylase, we co-expressed RHOA with HDAC family members (HDAC1-3) or SIRT family members (SIRT1-5), showing that RHOA lactylation was significantly reduced by HDAC3 but not by other HDAC or SIRT members (Fig. 5f and Supplementary Fig. 6 d). We also found that HDAC3 significantly reduced, but the inactive HDAC3-Y298F mutant did not affect, RHOA lactation levels (Fig. 5g). Significantly, knockdown of HDAC3 expression promoted the lactation levels of both K118 and K162 of RHOA (Fig. 5h). In vitro delactylation assay confirmed that HDAC3 could remove the lactylation of K118 and K162 of RHOA (Fig. 5i, j). Furthermore, exogenous HDAC3 expression enhanced RHOA ubiquitination (Fig. 5k). Clinical data analysis showed that patients with higher HDAC3 expression levels had shorter survival (Supplementary Fig. 6e). Together, these results suggest that HDAC3 is the major delactylase for RHOA.

### RHOA lactylation promotes metastasis in vivoand in vitro, and drugs inhibiting RHOA lactylation and targeting RHOA mutations can synergistically inhibit tumor cells

**Fig.6:**
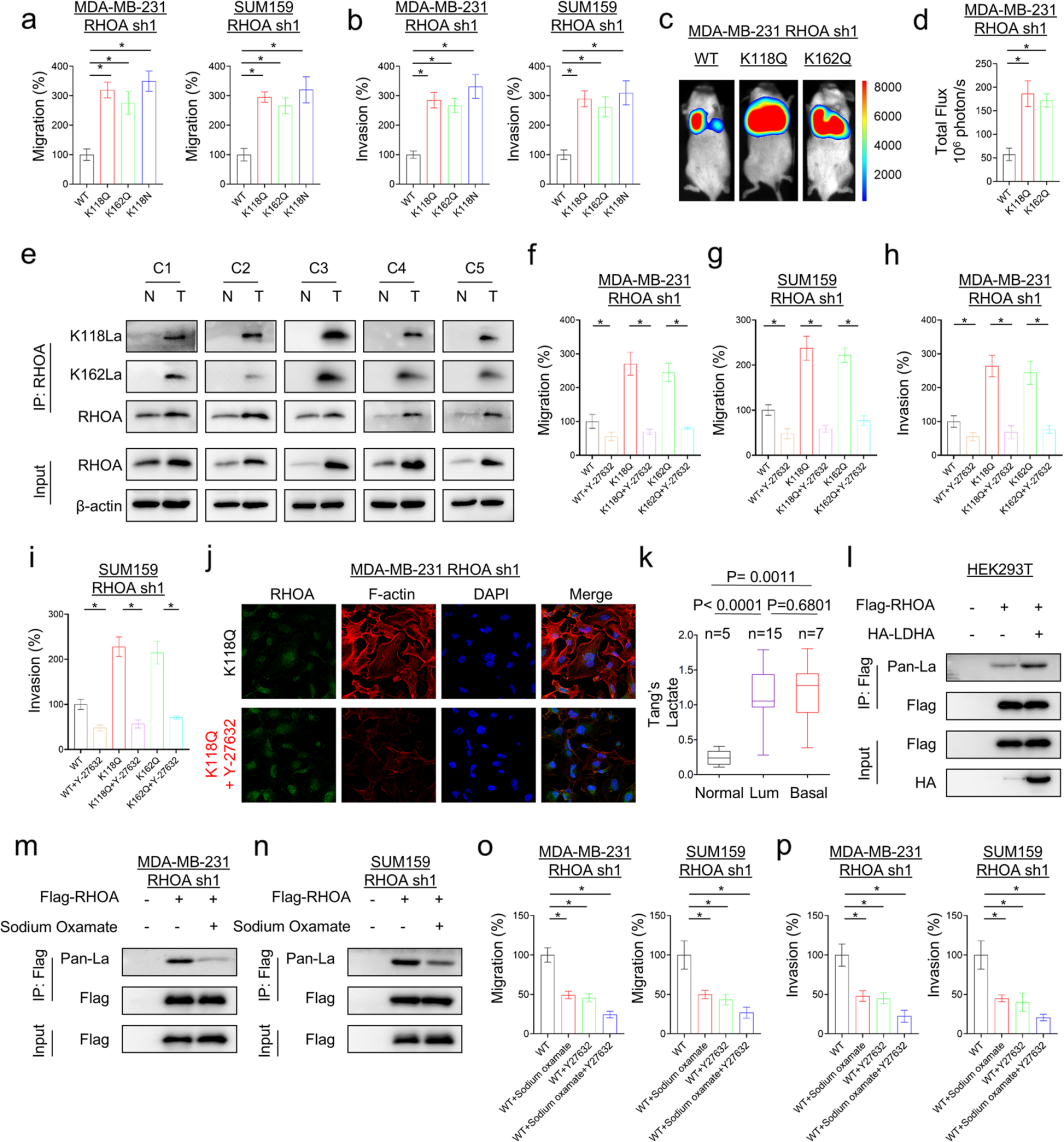
RHOA lactylation promotes metastasis in vivo and in vitro, and drugs inhibiting RHOA lactylation and targeting RHOA mutations can synergistically inhibit tumor cells. **a**, **b** Migratory ability (**a**) and invasiveness (**b**) of shRHOA-expressing MDA-MB231 and SUM159 cells with stable WT-RHOA, K118Q, K118N, or K162Q mutant were analyzed. The percentage of migratory and invasive cells was shown in the bar graph (mean ± SD in three separate experiments). *p < 0.05 by Student’s t-test. **c**, **d** shRHOA-expressing MDA-MB231 cells with stable WT-RHOA, K118Q or K162Q mutant expression were injected into nude mice via the tail vein. After 4 wks, the development of lung metastases was monitored using bioluminescence imaging (**c**) and quantified by measuring photon flux (mean of 6 animals + SEM) (**d**). Three representative mice from each group were shown (**c**). **e** Human breast tumor samples were paired with tumor tissue (designated as T) and adjacent normal tissue (designated as *N*), lysed and directly subjected to Western blotting for RHOA. For RHOA lactylation detection, tissue cell lysates were immunoprecipitated with RHOA antibody and then analyzed by Western blotting with site-specific antibody (K118la/K162la). **f**-**i** Migratory ability (**f**, **g**) and invasiveness (**h**, **i**) of shRHOA-expressing MDA-MB231 and SUM159 cells with stable WT-RHOA, K118Q or K162Q mutant expression were analyzed following treatment with or without Y-27632 (10 μM). The percentage of migratory and invasive cells was shown in the bar graph (mean ± SD in three separate experiments). *p < 0.05 by Student’s t-test. **j** F-actin was stained with Rhodamine Phalloidin (Red) in shRHOA-expressing MDA-MB231 cells with stable K118Q mutant expression following treatment with or without Y-27632 (10 μM). **k** Lactate level was analyzed in normal tissue, luminal, and basal-like tumors from Tang’s metabolomics dataset. **l** HEK293T cells were co-expressed with Flag-RHOA or/and HA-LDHA, followed by immunoprecipitation. The Kla levels of precipitated protein were analyzed by Western blot. **m**, **n** shRHOA-expressing MDA-MB231 (**m**) and SUM159 cells (**n**) were transfected with Flag-RHOA plasmid and cultured with or without treatment with sodium oxamate (20 mM) for 24 h, followed by immunoprecipitation. The Kla levels of precipitated protein were analyzed by Western blot. **o**, **p** Migratory ability (**o**) and invasiveness (**p**) of shRHOA-expressing MDA-MB231 and SUM159 cells with stable WT-RHOA expression were analyzed following treatment with Y-27632 (10 μM) or/and sodium oxamate (20 mM) for 24 h. The percentage of migratory and invasive cells was shown in the bar graph (mean ± SD in three separate experiments). *p < 0.05 by Student’s t-test

To reveal the potential biological functions of RHOA lactylation and mutations, RHOA lactylation-mimicking variant (K118Q and K162Q) and tumor-associated mutant variants (K118N) were ectopically expressed in multiple tumor cell lines. Compared with WT-RHOA, all K118Q, K118N and K162Q mutants led to increased cell colony formation, migration and invasion in vitro (Fig. 6a, b and Supplementary Fig. 7a). We also tested in vivo lung metastasis in a xenograft metastasis model. RHOA K118Q or K162Q caused a significant increase in lung metastasis in vivo compared with WT-RHOA (Fig. 6c, d and Supplementary Fig. 7b-d). We also generated RHOA-knockout (KO) cell lines and then reconstituted them with wild-type or mutant RHOA (Supplementary Fig. 7e). Compared to WT-reconstituted cells, RHOA-KO cells expressing the K118Q/K162Q mutants exhibited significantly enhanced metastasis (Supplementary Fig. 7f). In addition, we evaluated RHOA expression and lactylation in fresh breast cancer specimens and found that RHOA expression and lactylation were significantly upregulated in most paired breast cancer tissues compared with adjacent normal counterparts (Fig. 6e). Notably, the upregulation trend of RHOA lactylation in breast cancer tissues was even more obvious than that of RHOA (Fig. 6e). We then examined the effect of the ROCK inhibitor Y-27632 on RHOA mutant cell function in vitro. RHOA mutations cause a dramatic increase in breast cancer cell migration, invasion, and actin stress fiber formation, whereas Y-27632 significantly inhibited the effects of these increases (Fig. 6f-j and Supplementary Fig. 7 g). LDHA expression is significantly upregulated in different cancers and positively correlated with lactate levels. Our data showed that the lactate level in breast cancer tissues was significantly higher and the glucose level was significantly lower than that in normal breast tissues (Fig. 6k and Supplementary Fig. 7 h). Correlation analysis showed a positive correlation between LDHA and RHOA expression (Supplementary Fig. 7i). Consistently, LDHA expression significantly promoted, whereas the LDHA inhibitor sodium oxamate significantly inhibited, RHOA lactation levels (Fig. 6l-n), suggesting that LDHA inhibition may be a potential approach to treat patients with lactation-mediated RHOA activation. We then evaluated the effects of sodium oxamate and its combination with Y-27632 on tumor cell migration and invasion. Our results showed that sodium oxamate could significantly reduce tumor cell migration and invasion, and the effect was enhanced when combined with Y-27632, showing an obvious synergistic effect (Fig. 6o, p).

## Discussion

This study reports that RHOA lactylation at K118 and K162 enhances its activity and stability by mimicking oncogenic mutations, proposing lactylation as a reversible "epi-mutation" mechanism and revealing the critical role of RHOA lactylation in tumors (Fig. 5l).

### RHOA lactylation at sites prone to oncogenic mutations promotes tumor progression by mimicking mutational activation and stabilization

We identify a lactate-PCAF/HDAC3 axis regulating RHOA lactylation: PCAF catalyzes K118/K162 lactylation, whereas HDAC3 reverses this modification, linking the Warburg effect to RHOA activation. Notably, PCAF promoted, whereas AARS1 did not affect, RHOA lactylation, highlighting the substrate-dependent regulation of lactylation; HDAC3 acts as a metabolic checkpoint, dynamically balancing lactylation levels to modulate RHOA signaling in tumor microenvironments.

We demonstrated that lactylation and N/R mutation of K118, but not K162, rendered the RHOA protein in its GTP-bound active form. At K118 in the G4 motif (XKXD), the positively charged K118's ε-amino group normally forms hydrogen bonds with GTP's ribose [3, 20]. Therefore, the addition of a lactyl group to the ε-amino group of K118 may alter the GTP binding ability and GTPase activity of RHOA by eliminating the hydrogen bond with GTP. Although the compilation of RHOA crystal structures indicates that WT and K118-mutated RHOA have extremely similar conformations in GTP and its binding region, MD simulations showed that K118 lactylation-mimicking mutations or tumor-associated mutations weakened the hydrogen bonds associated with GTPase activity without affecting the binding flexibility of RHOA to GPT. Since the intracellular GTP concentration exceeds GDP by ~ tenfold [25], impaired hydrolysis shifts the equilibrium toward active GTP-RHOA, mimicking mutation-driven constitutive activation. Together, these data suggest that the functional consequences are twofold: (1)steric hindrance of GTP positioning in the binding pocket, and (2) disruption of the catalytic hydrogen bond network.

Several reports have shown that some proteins can be ubiquitinated on lysine residues that are often prone to oncogenic mutations [26, 27]. Our data identified that at K162 in the G5 motif (SAK), lactylation competitively blocked KCTD13-mediated K48-linked ubiquitination, reducing proteasomal degradation. This mechanism parallels cancer-associated K162N/R mutations, which similarly evade ubiquitination. USP9X amplified this effect via deubiquitination to stabilize RHOA. Thus, lactylation at both sites cooperatively enhances RHOA signaling: K118 increases activity, whereas K162 promotes protein stability.

Functionally, lactylated RHOA drives malignant phenotypes including enhanced migration and metastasis in vitro and in vivo. Clinically, RHOA lactylation levels are significantly elevated in breast cancer tissues compared to adjacent normal tissues. These findings establish RHOA lactylation as a critical metabolic regulatory mechanism that mimics oncogenic mutations to promote tumorigenesis.

In conclusion, our findings demonstrate that RHOA lactylation at K118 (activation) and K162 (stabilization) mimics oncogenic mutations. The PCAF/HDAC3 axis links lactate metabolism to RHOA signaling, revealing lactylation as a metabolic alternative to genetic mutations in cancer progression.

### Our study indicates a potential therapeutic strategy for tumors undergoing RHOA lactylation

LDHA-mediated lactate accumulation is tightly linked to protein lactylation [9–11]. Our data showed a positive correlation between lactate accumulation and RHOA lactylation and confirmed that the LDHA inhibitor oxamate inhibited the migration and invasion of cancer cells with RHOA lactylation, suggesting that inhibiting lactate production could be a potential therapeutic approach for patients with lactylated RHOA-driven tumors. Various approaches to target RHOA mutations have been explored, including inhibiting RHOA expression and downstream signal transduction [28, 29]. Our data found that the ROCK inhibitor Y27632 significantly inhibited cancer cells with lactation-mimicking variants and cancer-related mutations. Our further data showed that ROCK inhibitor and LDHA inhibitor had a better synergistic inhibitory effect. Collectively, due to the mechanistic and functional similarities between lactylation and mutations at the same site, lactylation-mediated RHOA activation may be treated individually or synergistically by inhibiting RHOA lactylation and targeting RHOA mutations.

### Our study proposes lactylation as a reversible "epi-mutation" system

We propose a paradigm-shifting concept wherein post-translational lactylation at mutation-prone sites functionally recapitulates same-site mutations, functioning as a reversible "epi-mutation" system. This metabolic paradigm enables mutation-equivalent oncoprotein activation while preserving genomic integrity, permitting rapid microenvironmental adaptation. Our findings challenge the traditional genetic/epigenetic dichotomy in cancer, explaining RHOA hyperactivity in mutation-negative tumors. Crucially, the use of a site-specific lactylation system overcomes mutagenesis artifacts, conclusively demonstrating that lactylation alone recapitulates mutational effects and validating both the biological mechanism and conceptual advance.

The concept may extend beyond RHOA and lactylation, other oncoproteins and PTMs likely employ similar "epi-mutation" mechanisms at shared regulatory sites. The discovery of this reversible mutation-mimicking system fundamentally expands our understanding of oncoprotein activation, revealing how tumors exploit metabolic modifications to achieve mutation-equivalent phenotypes without genetic changes. This has profound implications for precision oncology and therapeutic development.

In conclusion, our study establishes RHOA lactylation as a metabolic analog of oncogenic mutations, where lactylations at K118 (activation) and K162 (stabilization) drive tumor progression through constitutive RHOA signaling. The lactate-PCAF/HDAC3 axis links glycolytic metabolism to oncoprotein activation. Crucially, we reveal lactylation functions as a reversible "epi-mutation" system, dynamically mimicking genetic mutations while maintaining metabolic plasticity. Therapeutically, combined targeting of lactate metabolism and RHOA signaling represents a promising strategy for lactylation-driven cancers, bridging metabolic and genetic approaches in precision oncology.

### Limitations of the study

While our *site-specific lactylation system* enables precise incorporation of lactyl-lysine, its *transient expression* presents *limitations*. For *long-term* studies, *K-to-R mutations at K118/K162 were unsuitable* as controls due to intrinsic oncogenic effects, requiring *K-to-Q* substitutions that *mimic lactylation's polar characteristics* (*-NH2 for Q* vs *-CH3CHOH for lactylated K*) and *recapitulate lactylation's mechanistic and functional outcomes*. For *endogenous lactylation* detection in tumor tissues, *prior immunoprecipitation* using RHOA antibody *was required* to overcome *cross-reactivity* from conserved K118/K162 sites across RHO family proteins. These limitations underscore the necessity for improved PTM tools, yet our findings are substantiated by comprehensive validation incorporating site-specific lactylation, targeted mutagenesis, and molecular dynamics simulations.

## Supplementary Information

Supplementary Material 1: Supplementary Fig. 1. a-c, Kaplan-Meier survival analysis for OS (a), RFS (b) and DMFS (c) of patients in an aggregate breast cancer dataset according to RHOA expression status. The *p* value was determined using the log-rank test. d, e, SUM159 cells were transfected with Flag-RHOA plasmid and cultured in different concentrations of glucose (d) or sodium lactate (e) for 24 hours, followed by immunoprecipitation. The Kla levels of precipitated protein were analyzed by Western blot. f, g, MDA-MB231 (f) and SUM159 cells (g) were cultured in different concentrations of sodium lactate for 24 hours, followed by immunoprecipitation. The Kla levels of precipitated RHOA were analyzed by Western blot. h, Amino acid sequence logo of conserved G4 and G5 motifs in the RAS superfamily. i, j, WT-RHOA, K118R or K162R mutants were expressed in SUM159 cells, and lactylation of K118 (i) and K162 (j) was determined following immunoprecipitation. k, Kla was selectively incorporated into RHOA-K118 or K162TAG-EGFP in HEK293T cells using the Mb-Pyl Kla-RS/Pyl-tRNA pair; green fluorescence (bottom) and bright field (top) images were obtained to confirm the expression of EGFP in the absence or presence of 2 mM Kla. l, Following Kla was selectively incorporated into Flag-RHOA-K118 or K162TAG in HEK293T cells, expression of lactylated RHOA was analyzed by Western blotting. Supplementary Fig. 2. a, b, ROCK2-RBD-HA was co-expressed with WT (WT), lactylated (a), or different mutant RHOA (b) in HEK293T cells, and the immunoprecipitated ROCK2-RBD-HA was examined by Western blotting. c, Purified GST and GST-RBD were detected by Coomassie blue staining. d, WT-RHOA or different mutants were expressed in HEK293T cells, and their activities were determined by GST-RBD pull-down assay. e, Expression of p-MLC2 and MLC2 in HEK293T with WT-RHOA or lactylated RHOA was examined by Western blotting. f, Stable knockdown of RHOA expression was established in MDA-MB231 and SUM159 cells. RHOA expression in these cells was analyzed by Western blotting. Actin was used as a loading control. g, Relative fluorescence intensity of F-actin in shRHOA-expressing MDA-MB-231 cells with stable WT-RHOA or K118Q mutant expression was analyzed usign ImageJ. h, F-actin was stained with Rhodamine Phalloidin (Red) in shRHOA-expressing SUM159 cells with stable WT-RHOA or K118Q mutant expression. i, Structural illustration of lysine lactylation, lysine acetylation and glutamine. Supplementary Fig.3. a, Interactions of K118 within the G4 motif and K162 within the G5 motif with GTP-bound RHOA (PDB: 4XOI). Hydrogen bond interactions are shown as dashed lines. b, The correlation of K118 and K162 with GTP in the RHOA is represented by the crystal structure (PDB: 1FTN). c, Overlay of the WT-RHOA crystal structure (PDB: 1FTN; tan) and modeled WT-RHOA (sky blue). A magnified overlay of GDP and GTP structure was shown on the right. d, Overlay of WT-RHOA (tan) with K118Q (sky blue), K118R (pink), and K118N mutants (light green), respectively after modeling. A magnified overlay of GTP structure was shown on the right. e-g, Overlay and histogram for protein backbone Cα RMSF plots of MD trajectories between WT-RHOA and K118Q (e), K118R (f), or K118N mutant (g). h, The binding activity between GTP and purified RHOA, RHOA mutant (K118Q), or RHOA-K118La (from site-specific glutarylation system) were determined by GTP pull-down assay. i, Heatmap for hydrogen-bonding interactions of GTP with WT-RHOA, K118Q, K118R, or K118N mutant. Occupancy is defined as the ratio of the simulation time that a particular hydrogen bond is present. Supplementary Fig. 4. a, MS/MS spectra of representative RHOA peptides carrying K118 ubiquitination. b, WT-RHOA was expressed in HEK293T cells for 48 h, and then the cells were treated with or without 10 μM MG132 for 6 h. Cell lysates were analyzed by Western blotting. c, For CHX assay, shRHOA-expressing SUM159 cells with WT-RHOA or K162 mutant expression were treated with 25 μg/mL CHX for the indicated time; the expression level of RHOA was examined by Western blotting. d, Flag-RHOA was co-transfected with HA-ubiquitin into HEK293T cells. Ubiquitination of immunoprecipitated RHOA was measured. e, HEK293T cells were co-transfected with the indicated plasmids, ubiquitination of K118R mutant was measured by ubiquitination assay. f, Box-plots indicate KCTD13 mRNA expression in different subtypes of breast cancer from two datasets (MEBTABRIC and GSE25066). g, Analysis of GSE25066 dataset for the relationship between KCTD13 expression and chemotherapy sensitivity. h-j, Kaplan-Meier survival analysis for RFS, and DMFS of patients in the aggregate breast cancer dataset and the GSE25066 dataset according to KCTD13 expression status. The *p* value was determined using the log-rank test. k, The lysates of HEK293T cells with WT-RHOA, K162R mutant, or/and KCTD13 expression were subjected to immunoprecipitation with Flag-M2 agarose, and then their interactions were analyzed by Western blotting. Supplementary Fig. 5. a, Table presenting a portion of the RHOA-binding proteins identified through proximity labeling mass spectrometry (PL-MS). b, Box-plots indicate USP9X expression in different histological grades of breast cancer from the MEBTABRIC dataset. c-e, Kaplan-Meier survival analysis for OS, RFS, and DMFS of patients in the indicated datasets according to USP9X expression status. The *p* value was determined using the log-rank test. f, HEK293T cells were co-transfected with the indicated plasmids, ubiquitination of RHOA was measured by ubiquitination assay. Supplementary Fig. 6 a, b, In vitro lactylation assays were used to examine the effect of PCAF on the lactylation of wild-type RHOA-K118 (a) and RHOA-K162 (b), and their corresponding site mutants. c, HEK293T cells were transfected with RHOA and/or AARS plasmids, and the lactylation level of RHOA was measured using a pan-Kla antibody. d, HEK293T cells were co-expressed with RHOA, and indicated SIRT, and the lactylation level of RHOA was measured using a pan-Kla antibody. e, Kaplan-Meier survival analysis of patients in the indicated datasets according to HDAC3 mRNA expression status. The *p* value was determined using the log-rank test. Supplementary Fig. 7. a, The formation of colonies from shRHOA-expressing MDA-MB231 and SUM159 cells with stable WT-RHOA, K118Q, or K162Q mutant was measured. Data were presented as a percentage of empty vector cell lines (mean ± SD in three separate experiments). *p< 0.01 by Student’s t-test. b, shRHOA-expressing MDA-MB231 cells with stable WT-RHOA, K118Q or K162Q mutant expression were injected into nude mice via the tail vein. After 4 wks, the development of lung metastases was examined in paraffin-embedded sections stained with hematoxylin and eosin. c, d, Ki67 was analyzed by immunohistochemistry in the indicated tumor tissues from nude mice (mean ± SD in three separate experiments). e, RHOA was successfully knocked out in MDA-MB231 cells (left). WT-RHOA, K118Q, or K162Q mutants were stably transfected into the RHOA-knockout MDA-MB231 cells (right). Western blotting was used to analyze RHOA expression in these cells. Actin was used as a loading control. f, RHOA-knockout MDA-MB231 cells with stable WT-RHOA, K118Q or K162Q mutant expression were injected into nude mice via the tail vein. After 4 wks, the development of lung metastases was monitored using bioluminescence imaging (left) and quantified by measuring photon flux (mean of 6 animals + SEM) (right). Three representative mice from each group were shown (left). g, F-actin was stained with Rhodamine Phalloidin (Red) in shRHOA-expressing SUM159 cells with stable K118Q mutant expression following treatment with or without Y-27632 (10 μM). h, Glucose level was analyzed in normal tissue, luminal, and basal-like tumors from Tang’s metabolomics dataset. i, Analysis of Johansson’s dataset for the expression of RHOA and LDHA. The relative level of RHOA was plotted against that of LDHA.

## Data Availability

No datasets were generated or analysed during the current study.
