# Supplementary material for: RHOA lactylation at oncogenic hotspots promotes oncogenic activity and protein stabilization
Source: Mol Cancer. 2025 Nov 25;24:311. doi: 10.1186/s12943-025-02511-7 (PMC12750741; doi:10.1186/s12943-025-02511-7)

Fig 1

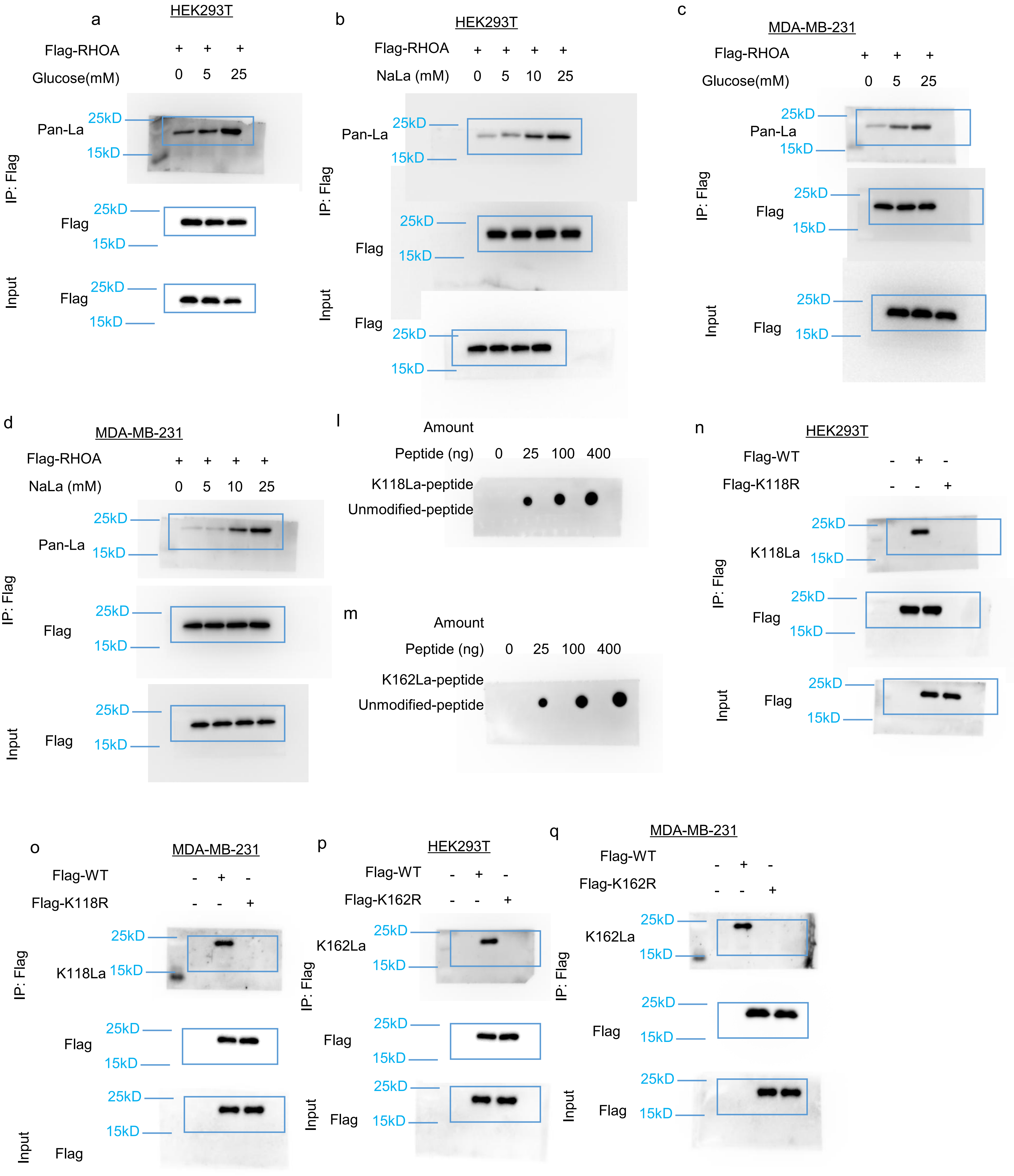
a
HEK293T
Flag-RHOA + + +
Glucose(mM) 0 5 25
Pan-La
25kD
15kD
Flag
25kD
15kD
IP: Flag
Input
Flag
25kD
15kD
b
HEK293T
Flag-RHOA + + + +
NaLa (mM) 0 5 10 25
Pan-La
25kD
15kD
Flag
25kD
15kD
IP: Flag
Input
Flag
25kD
15kD
c
MDA-MB-231
Flag-RHOA + + +
Glucose(mM) 0 5 25
Pan-La
25kD
15kD
Flag
25kD
15kD
IP: Flag
Input
Flag
25kD
15kD
d
MDA-MB-231
Flag-RHOA + + + +
NaLa (mM) 0 5 10 25
Pan-La
25kD
15kD
Flag
25kD
15kD
IP: Flag
Input
Flag
25kD
15kD
l
Amount
Peptide (ng) 0 25 100 400
K118La-peptide
Unmodified-peptide
m
Amount
Peptide (ng) 0 25 100 400
K162La-peptide
Unmodified-peptide
n
HEK293T
Flag-WT - + -
Flag-K118R - - +
K118La
25kD
15kD
Flag
25kD
15kD
IP: Flag
Input
Flag
25kD
15kD
o
MDA-MB-231
Flag-WT - + -
Flag-K118R - - +
K118La
25kD
15kD
Flag
25kD
15kD
IP: Flag
Input
Flag
25kD
15kD
p
HEK293T
Flag-WT - + -
Flag-K162R - - +
K162La
25kD
15kD
Flag
25kD
15kD
IP: Flag
Input
Flag
25kD
15kD
q
MDA-MB-231
Flag-WT - + -
Flag-K162R - - +
K162La
25kD
15kD
Flag
25kD
15kD
IP: Flag
Input
Flag
25kD
15kD

Fig 1

s

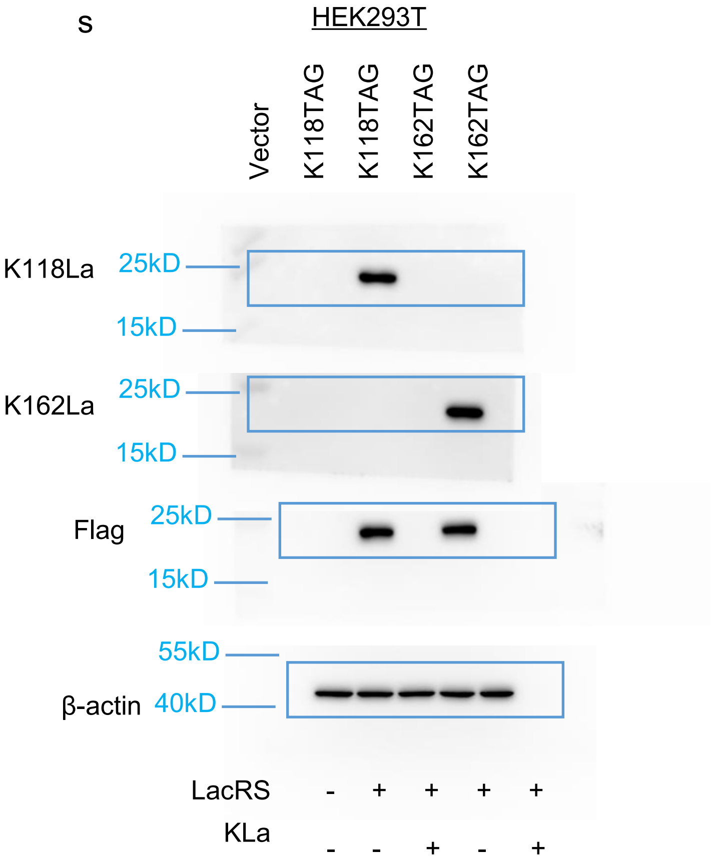

HEK293T
Vector
K118TAG
K118TAG
K162TAG
K162TAG
K118La
25kD
15kD
K162La
25kD
15kD
Flag
25kD
15kD
55kD
β-actin
40kD
LacRS - + + + +
KLa - - + - +

Fig 2

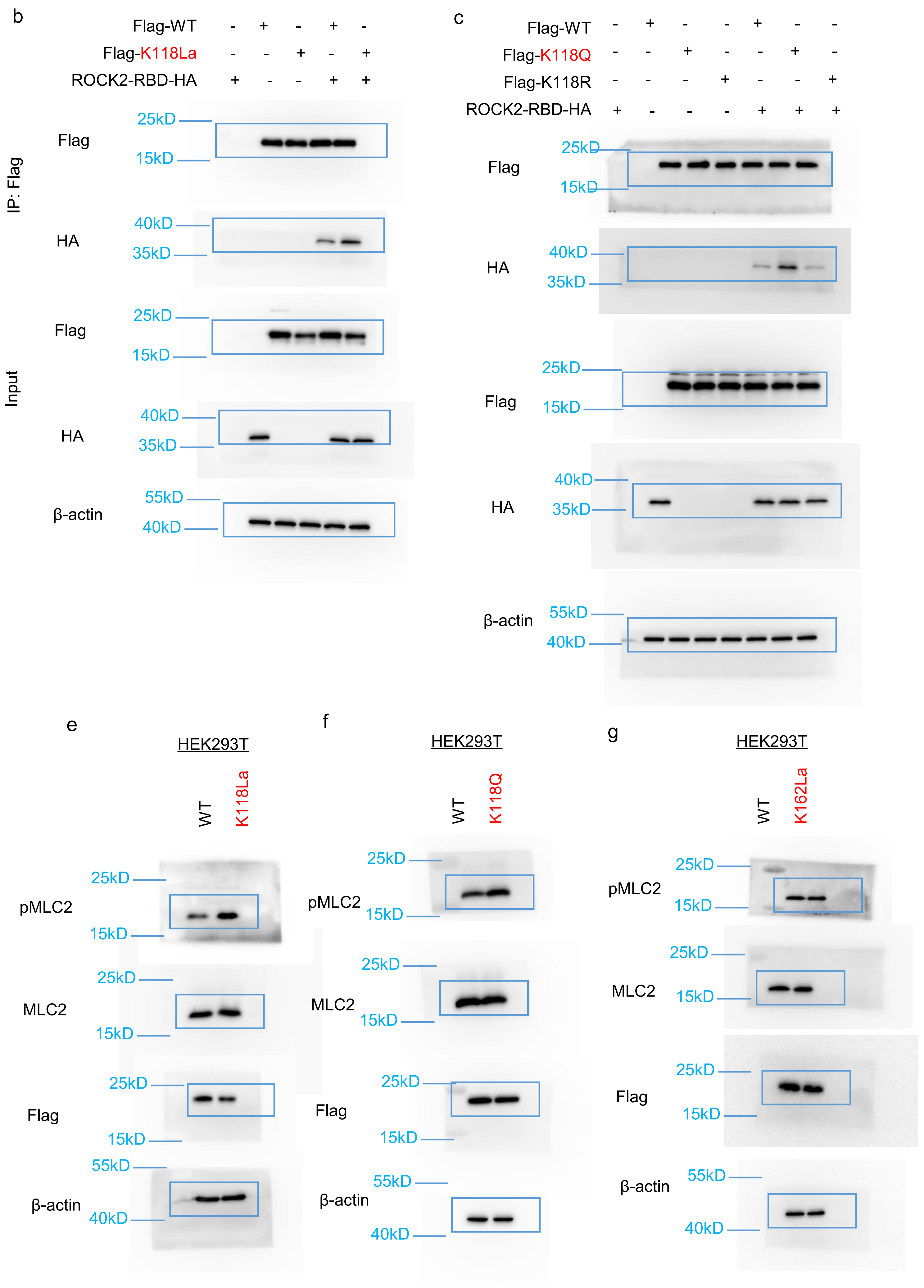

b
Flag-WT - + - + -
Flag-K118La - - + - +
ROCK2-RBD-HA + - - + +
IP: Flag
Flag
25kD
15kD
HA
40kD
35kD
Input
Flag
25kD
15kD
HA
40kD
35kD
β-actin
55kD
40kD
c
Flag-WT - + - - + - -
Flag-K118Q - - + - - + -
Flag-K118R - - - + - - +
ROCK2-RBD-HA + - - - + + +
Flag
25kD
15kD
HA
40kD
35kD
Flag
25kD
15kD
HA
40kD
35kD
β-actin
55kD
40kD
e
HEK293T
WT
K118La
pMLC2
25kD
15kD
MLC2
25kD
15kD
Flag
25kD
15kD
β-actin
55kD
40kD
f
HEK293T
WT
K118Q
pMLC2
25kD
15kD
MLC2
25kD
15kD
Flag
25kD
15kD
β-actin
55kD
40kD
g
HEK293T
WT
K162La
pMLC2
25kD
15kD
MLC2
25kD
15kD
Flag
25kD
15kD
β-actin
55kD
40kD

Fig 2

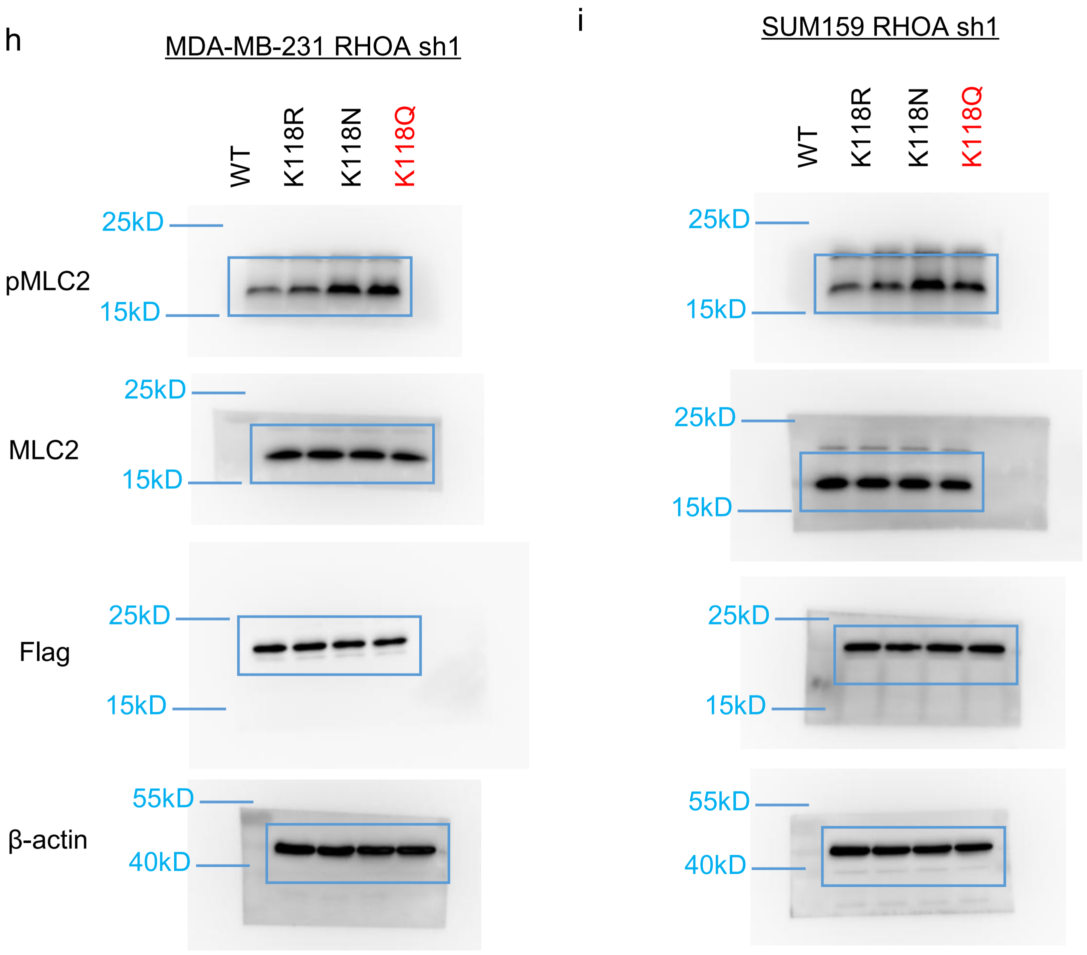

h
MDA-MB-231 RHOA sh1
WT
K118R
K118N
K118Q
pMLC2
MLC2
Flag
β-actin
25kD
15kD
55kD
40kD
i
SUM159 RHOA sh1
WT
K118R
K118N
K118Q
25kD
15kD
55kD
40kD

Fig 3

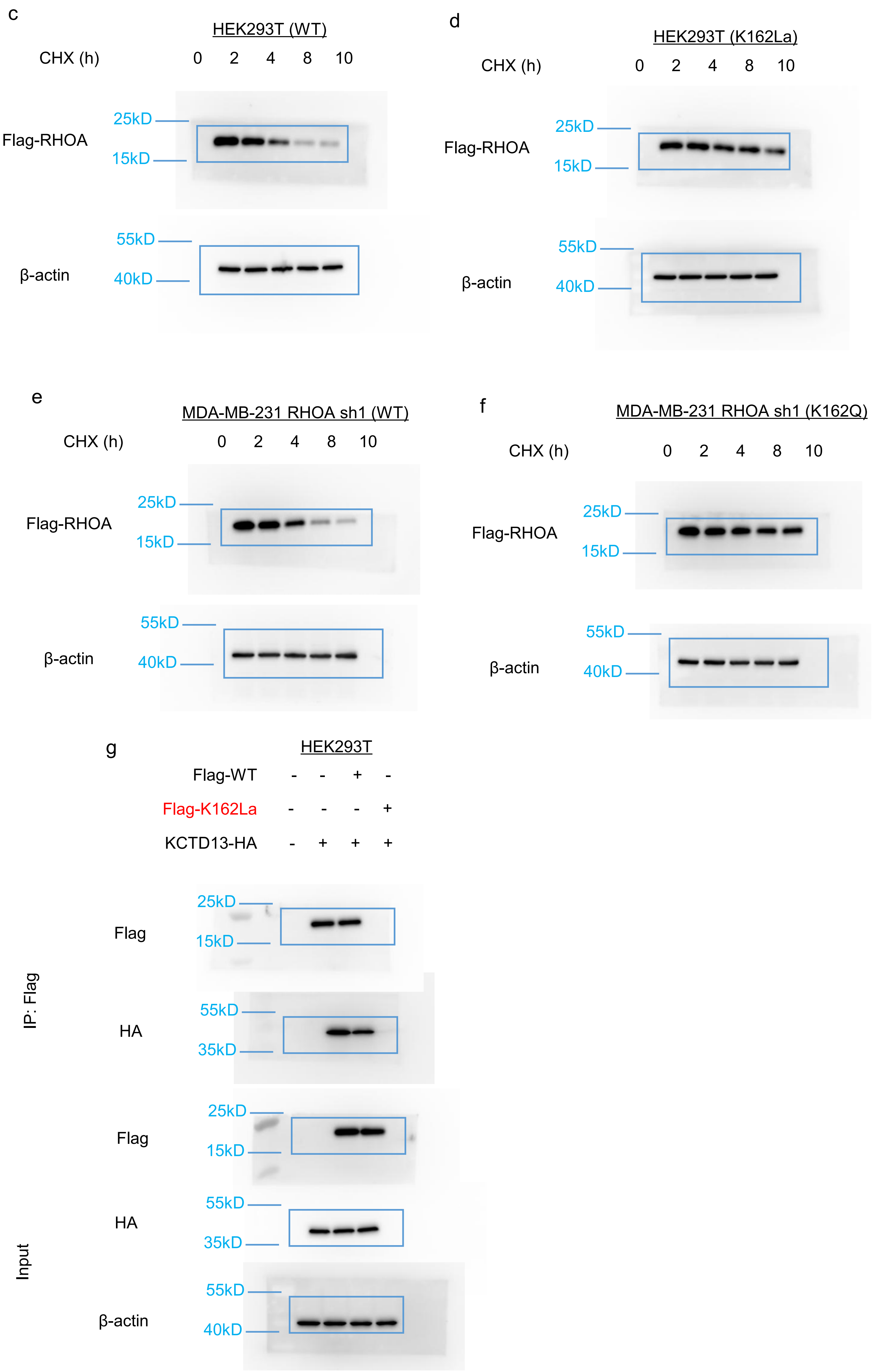
c
HEK293T (WT)
CHX (h)
0 2 4 8 10
Flag-RHOA
25kD
15kD
β-actin
55kD
40kD
d
HEK293T (K162La)
CHX (h)
0 2 4 8 10
Flag-RHOA
25kD
15kD
β-actin
55kD
40kD
e
MDA-MB-231 RHOA sh1 (WT)
CHX (h)
0 2 4 8 10
Flag-RHOA
25kD
15kD
β-actin
55kD
40kD
f
MDA-MB-231 RHOA sh1 (K162Q)
CHX (h)
0 2 4 8 10
Flag-RHOA
25kD
15kD
β-actin
55kD
40kD
g
HEK293T
Flag-WT - - + -
Flag-K162La - - - +
KCTD13-HA - + + +
IP: Flag
Flag
25kD
15kD
HA
55kD
35kD
Input
Flag
25kD
15kD
HA
55kD
35kD
β-actin
55kD
40kD

Fig 3

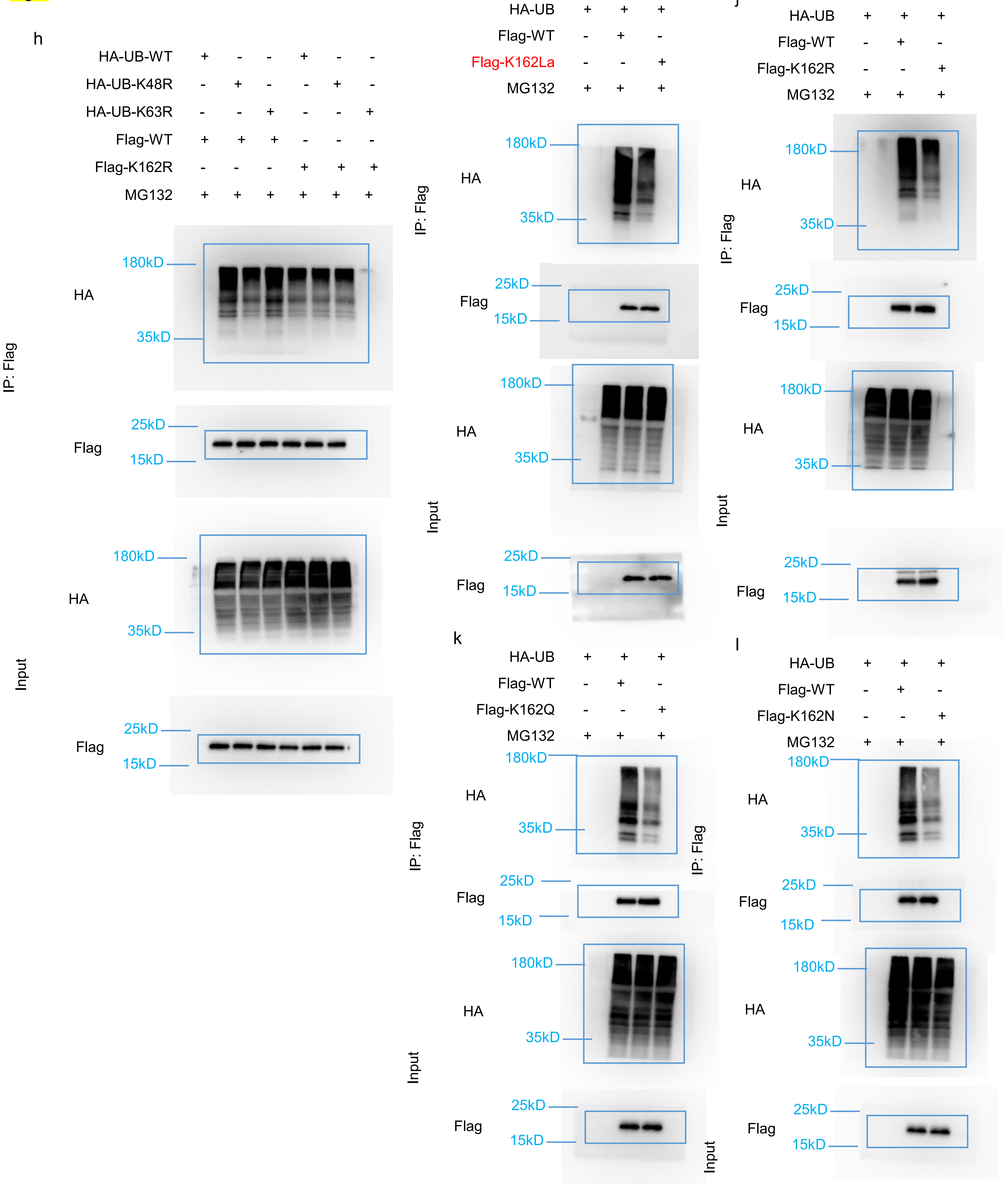
h
HA-UB-WT + - - + - -
HA-UB-K48R - + - - + -
HA-UB-K63R - - + - - +
Flag-WT + + + - - -
Flag-K162R - - - + + +
MG132 + + + + + +
IP: Flag
HA
180kD
35kD
Flag
25kD
15kD
Input
HA
180kD
35kD
Flag
25kD
15kD
i
HA-UB + + +
Flag-WT - + -
Flag-K162La - - +
MG132 + + +
IP: Flag
HA
180kD
35kD
Flag
25kD
15kD
Input
HA
180kD
35kD
Flag
25kD
15kD
j
HA-UB + + +
Flag-WT - + -
Flag-K162R - - +
MG132 + + +
IP: Flag
HA
180kD
35kD
Flag
25kD
15kD
Input
HA
180kD
35kD
Flag
25kD
15kD
k
HA-UB + + +
Flag-WT - + -
Flag-K162Q - - +
MG132 + + +
IP: Flag
HA
180kD
35kD
Flag
25kD
15kD
Input
HA
180kD
35kD
Flag
25kD
15kD
l
HA-UB + + +
Flag-WT - + -
Flag-K162N - - +
MG132 + + +
IP: Flag
HA
180kD
35kD
Flag
25kD
15kD
Input
HA
180kD
35kD
Flag
25kD
15kD

Fig 4

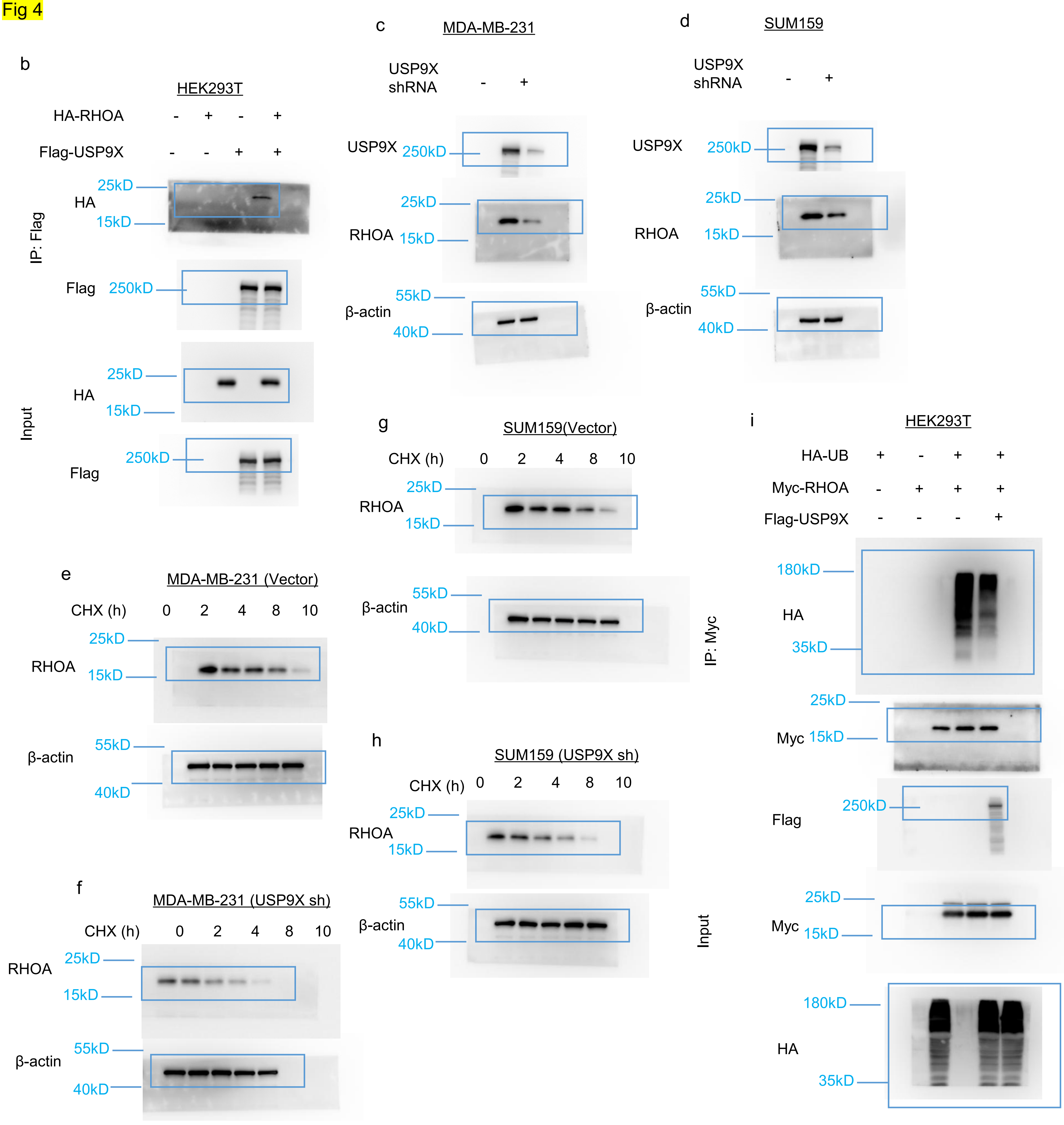
b
HEK293T
HA-RHOA - + - +
Flag-USP9X - - + +
IP: Flag
HA
25kD
15kD
Flag
250kD
Input
HA
25kD
15kD
Flag
250kD
c
MDA-MB-231
USP9X shRNA - +
USP9X
250kD
RHOA
25kD
15kD
β-actin
55kD
40kD
d
SUM159
USP9X shRNA - +
USP9X
250kD
RHOA
25kD
15kD
β-actin
55kD
40kD
e
MDA-MB-231 (Vector)
CHX (h) 0 2 4 8 10
RHOA
25kD
15kD
β-actin
55kD
40kD
f
MDA-MB-231 (USP9X sh)
CHX (h) 0 2 4 8 10
RHOA
25kD
15kD
β-actin
55kD
40kD
g
SUM159(Vector)
CHX (h) 0 2 4 8 10
RHOA
25kD
15kD
β-actin
55kD
40kD
h
SUM159 (USP9X sh)
CHX (h) 0 2 4 8 10
RHOA
25kD
15kD
β-actin
55kD
40kD
i
HEK293T
HA-UB + - + +
Myc-RHOA - + + +
Flag-USP9X - - - +
IP: Myc
HA
180kD
35kD
Myc
25kD
15kD
Input
Flag
250kD
Myc
25kD
15kD
HA
180kD
35kD

Fig 4

l

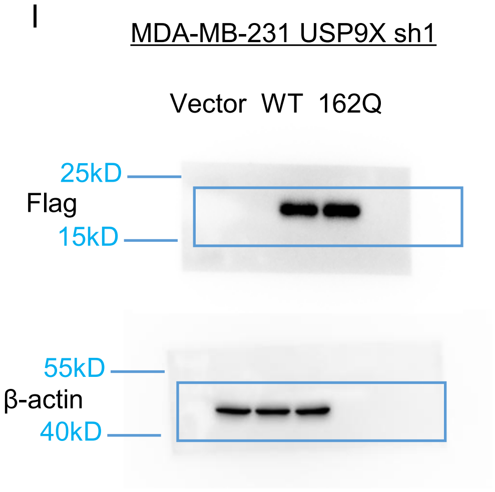
MDA-MB-231 USP9X sh1
Vector WT 162Q
25kD
Flag
15kD
55kD
β-actin
40kD

n

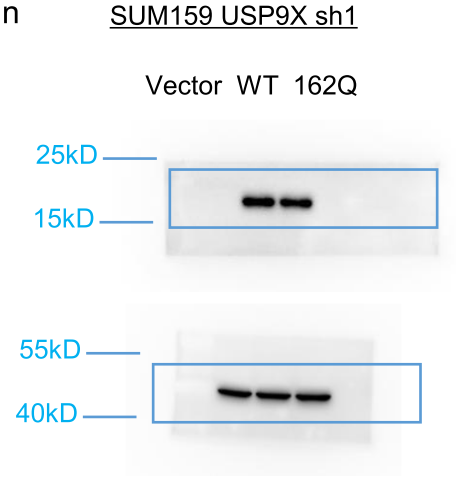
SUM159 USP9X sh1
Vector WT 162Q
25kD
15kD
55kD
40kD

Fig 5

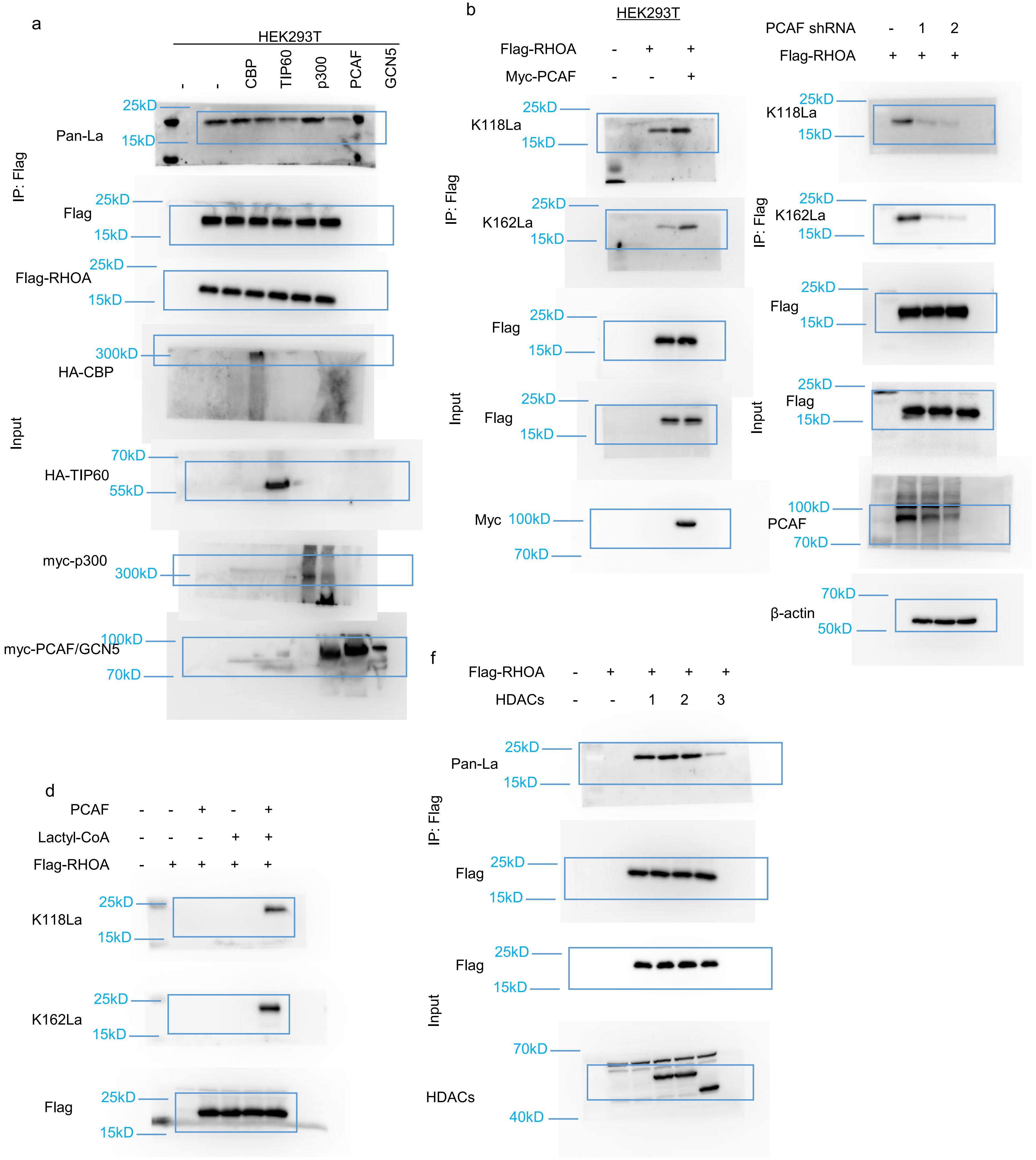
a
HEK293T
-
-
CBP
TIP60
p300
PCAF
GCN5
IP: Flag
Pan-La
25kD
15kD
Flag
25kD
15kD
Input
Flag-RHOA
25kD
15kD
HA-CBP
300kD
HA-TIP60
70kD
55kD
myc-p300
300kD
myc-PCAF/GCN5
100kD
70kD
b
HEK293T
Flag-RHOA - + +
Myc-PCAF - - +
IP: Flag
K118La
25kD
15kD
K162La
25kD
15kD
Flag
25kD
15kD
Input
Flag
25kD
15kD
Myc
100kD
70kD
c
PCAF shRNA - 1 2
Flag-RHOA + + +
IP: Flag
K118La
25kD
15kD
K162La
25kD
15kD
Flag
25kD
15kD
Input
Flag
25kD
15kD
PCAF
100kD
70kD
β-actin
70kD
50kD
d
PCAF - - + - +
Lactyl-CoA - - - + +
Flag-RHOA - + + + +
K118La
25kD
15kD
K162La
25kD
15kD
Flag
25kD
15kD
f
Flag-RHOA - + + + +
HDACs - - 1 2 3
IP: Flag
Pan-La
25kD
15kD
Flag
25kD
15kD
Input
Flag
25kD
15kD
HDACs
70kD
40kD

Fig 5

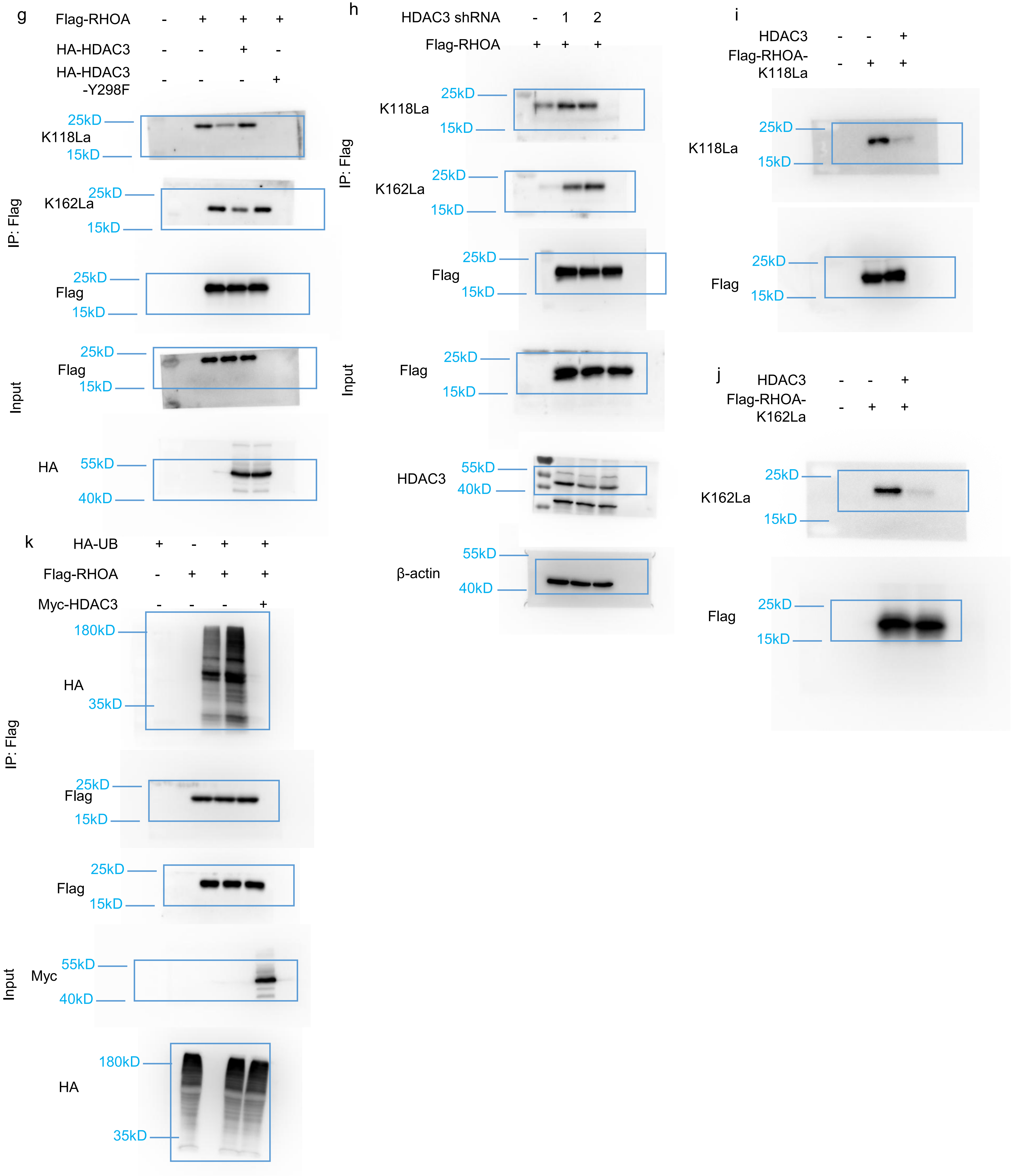

g
Flag-RHOA - + + +
HA-HDAC3 - - + -
HA-HDAC3 -Y298F - - - +
IP: Flag
25kD
K118La
15kD
25kD
K162La
15kD
25kD
Flag
15kD
Input
25kD
Flag
15kD
HA
55kD
40kD
h
HDAC3 shRNA - 1 2
Flag-RHOA + + +
IP: Flag
25kD
K118La
15kD
25kD
K162La
15kD
25kD
Flag
15kD
Input
Flag
25kD
15kD
HDAC3
55kD
40kD
β-actin
55kD
40kD
i
HDAC3 - - +
Flag-RHOA-K118La - + +
25kD
K118La
15kD
25kD
Flag
15kD
j
HDAC3 - - +
Flag-RHOA-K162La - + +
25kD
K162La
15kD
25kD
Flag
15kD
k
HA-UB + - + +
Flag-RHOA - + + +
Myc-HDAC3 - - - +
IP: Flag
180kD
HA
35kD
25kD
Flag
15kD
25kD
Flag
15kD
Input
Myc
55kD
40kD
HA
180kD
35kD

Fig 6

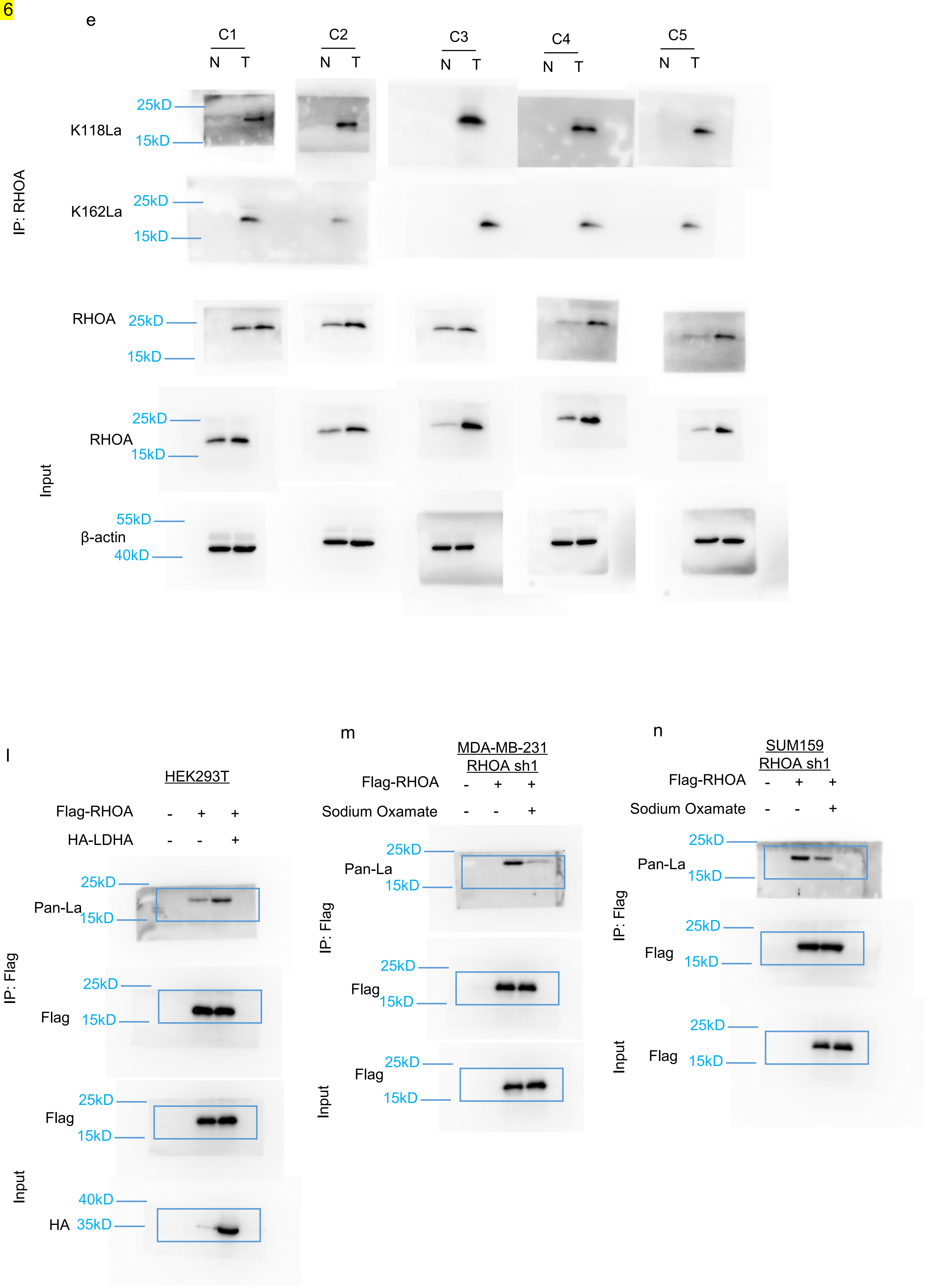

e
C1
C2
C3
C4
C5
N T
IP: RHOA
K118La
K162La
RHOA
Input
RHOA
β-actin
25kD
15kD
55kD
40kD
l
HEK293T
Flag-RHOA - + +
HA-LDHA - - +
IP: Flag
Pan-La
Flag
Input
Flag
HA
40kD
35kD
m
MDA-MB-231
RHOA sh1
Flag-RHOA - + +
Sodium Oxamate - - +
IP: Flag
Pan-La
Flag
Input
Flag
n
SUM159
RHOA sh1
Flag-RHOA - + +
Sodium Oxamate - - +
IP: Flag
Pan-La
Flag
Input
Flag

Supplementary Fig 1

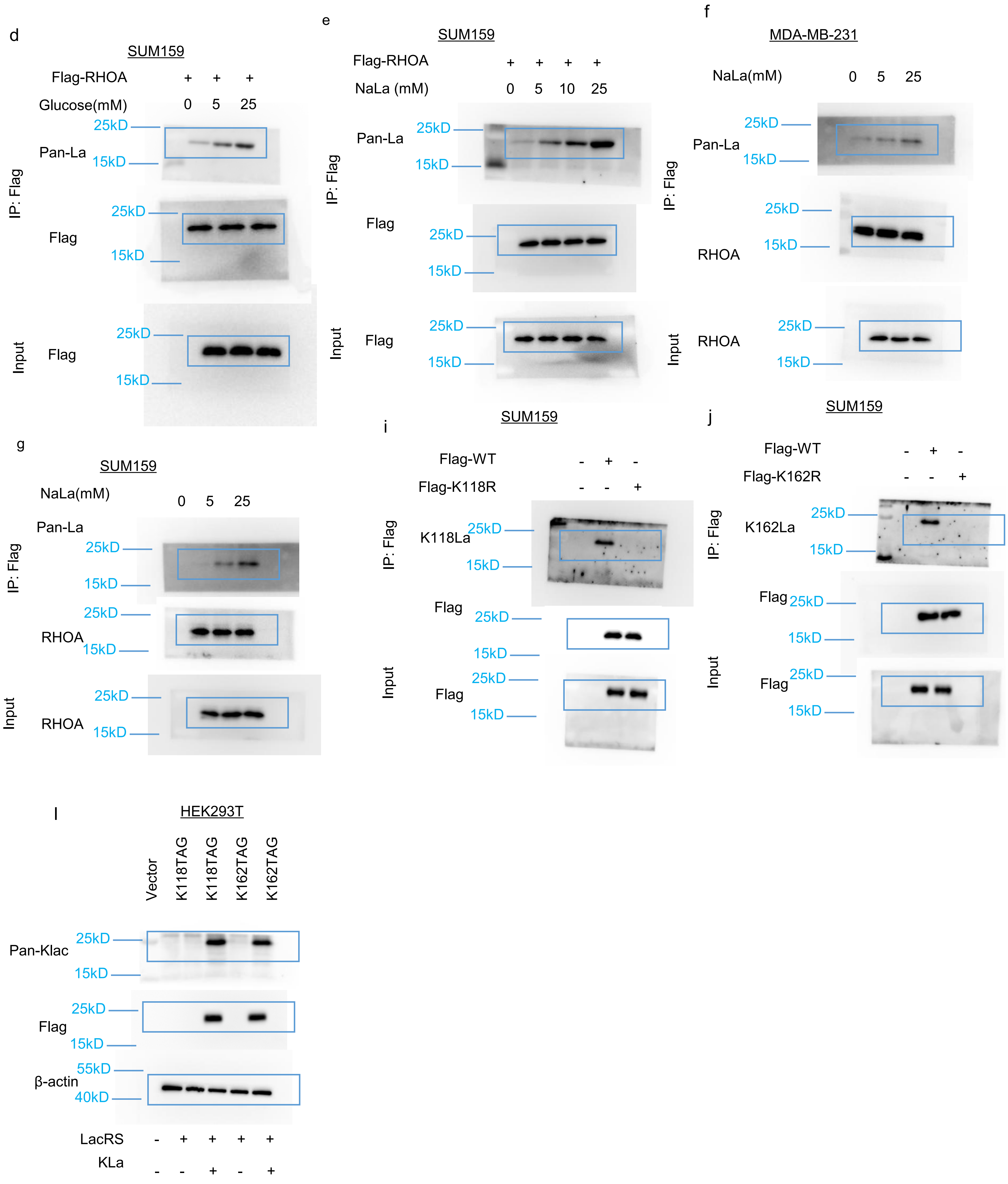

Supplementary Fig 2

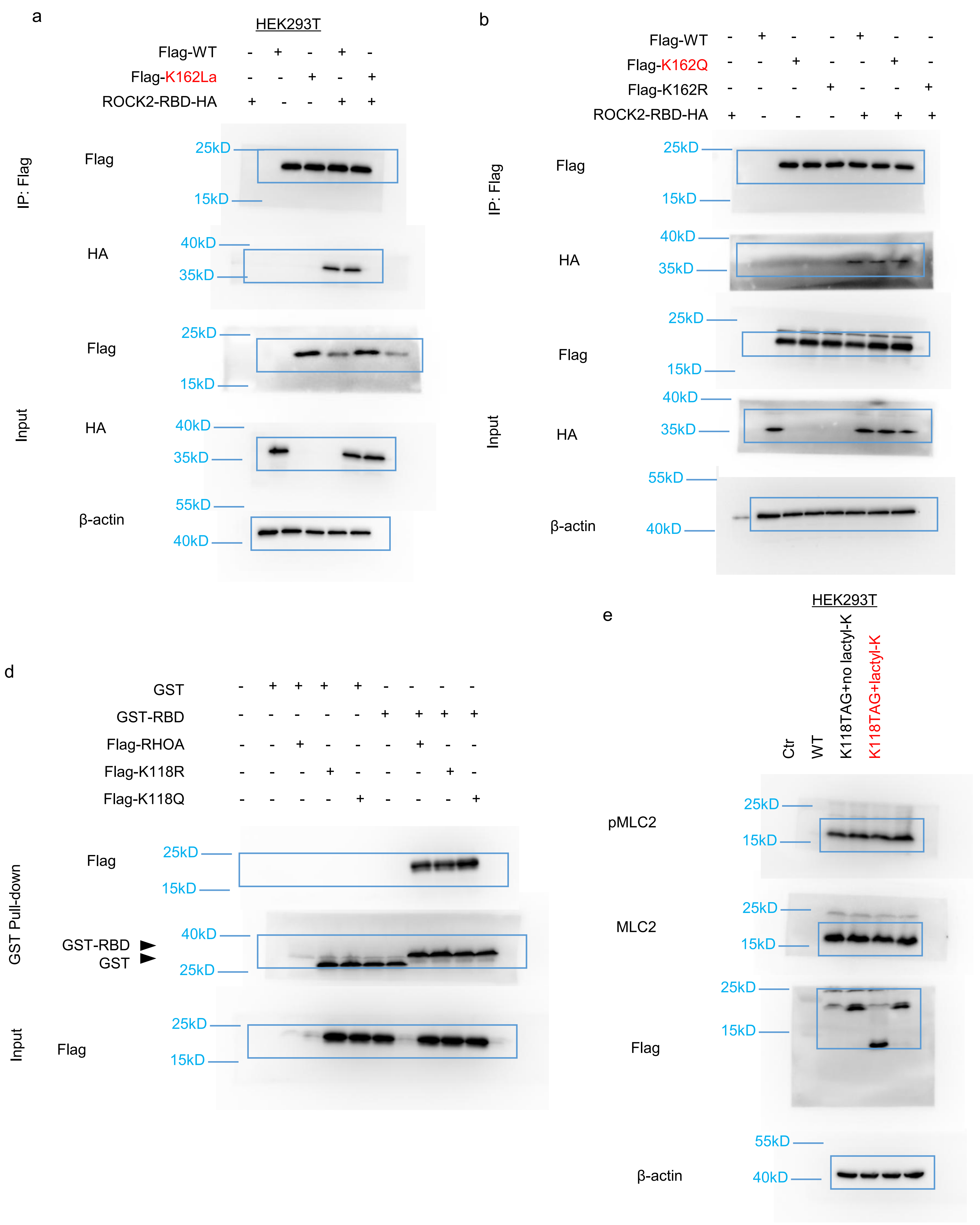

Supplementary Fig 2

f

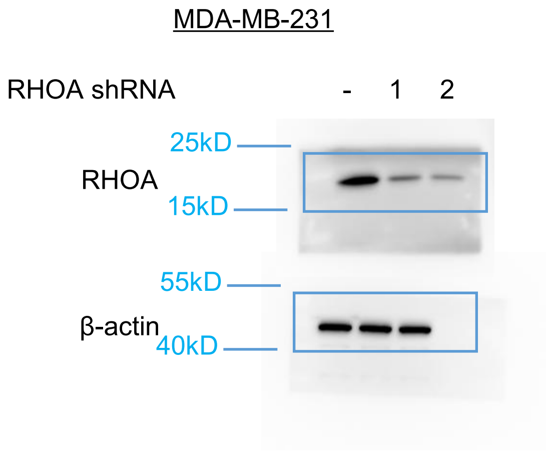

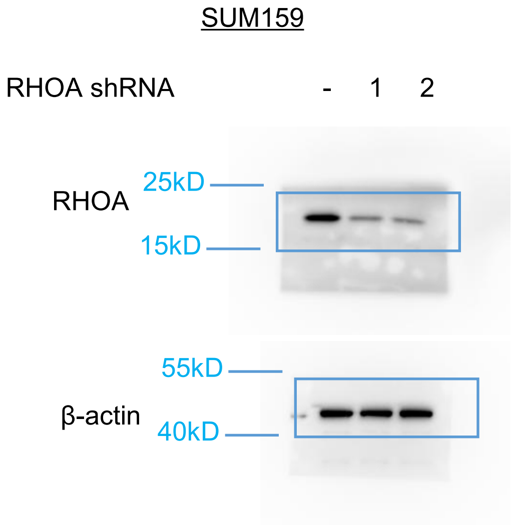

Supplementary Fig 3

g

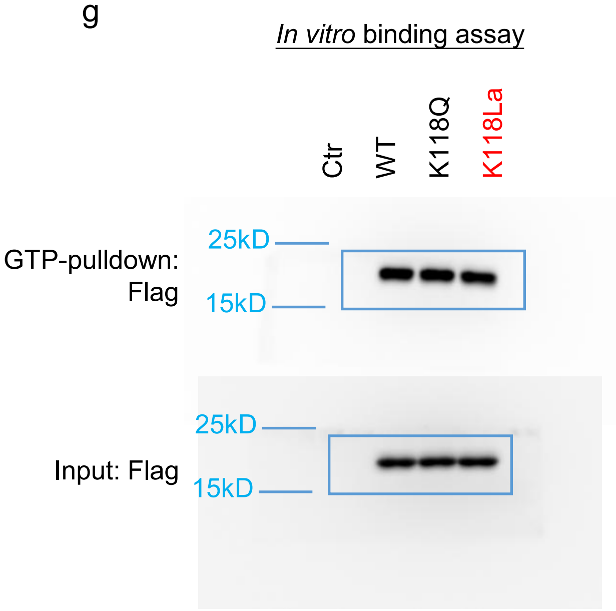

Supplementary Fig 4

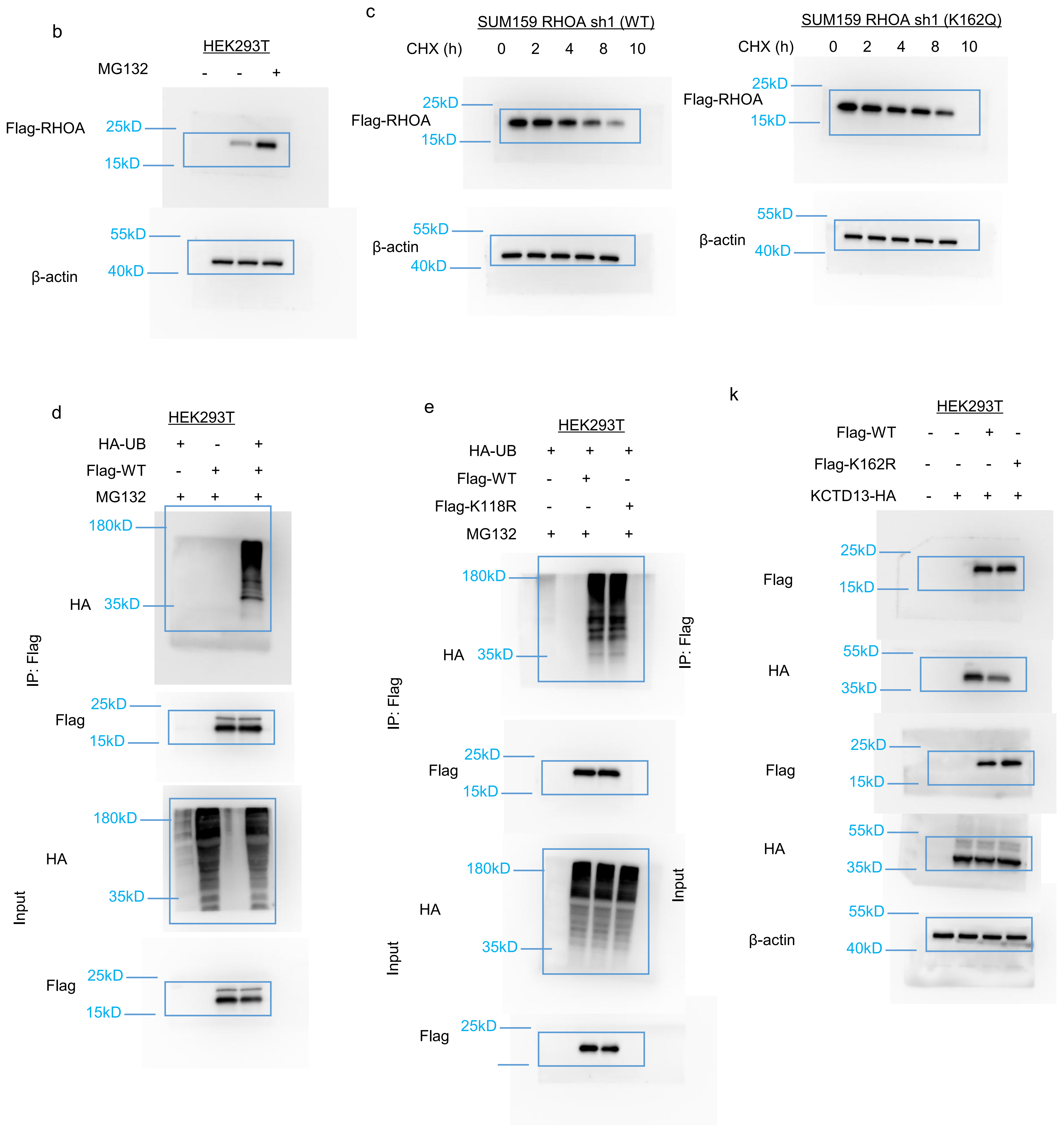

Supplementary Fig 5

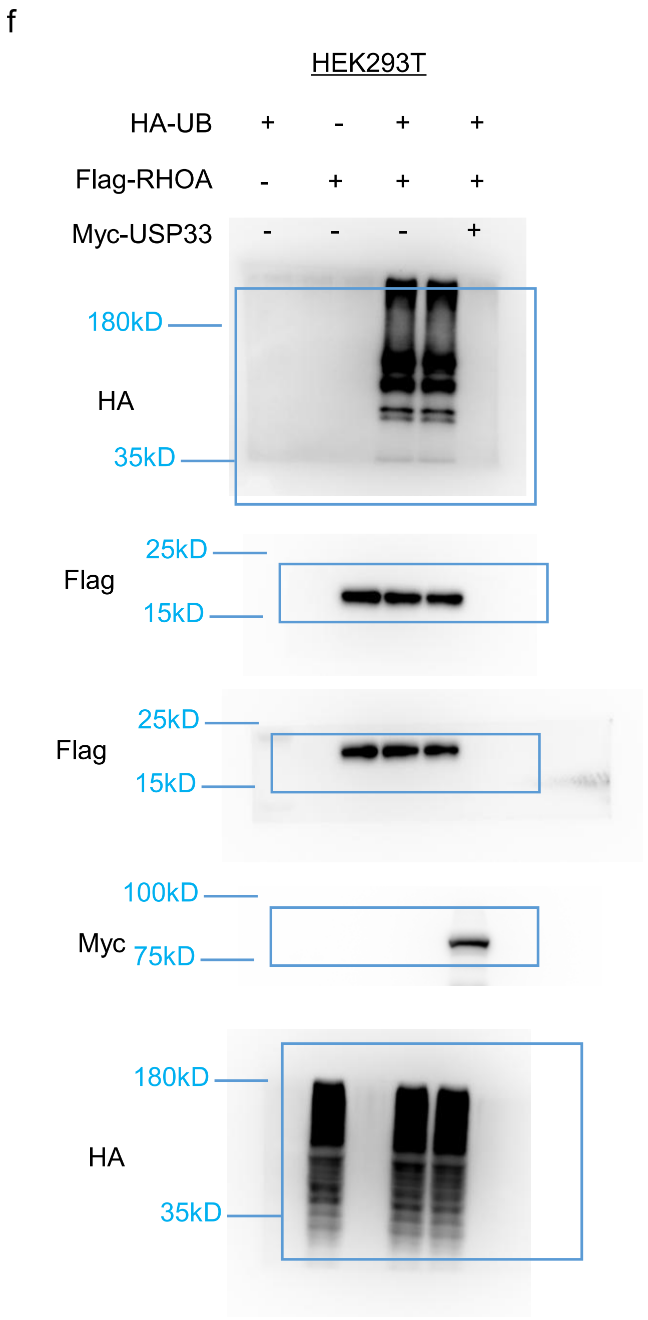

Supplementary Fig 6

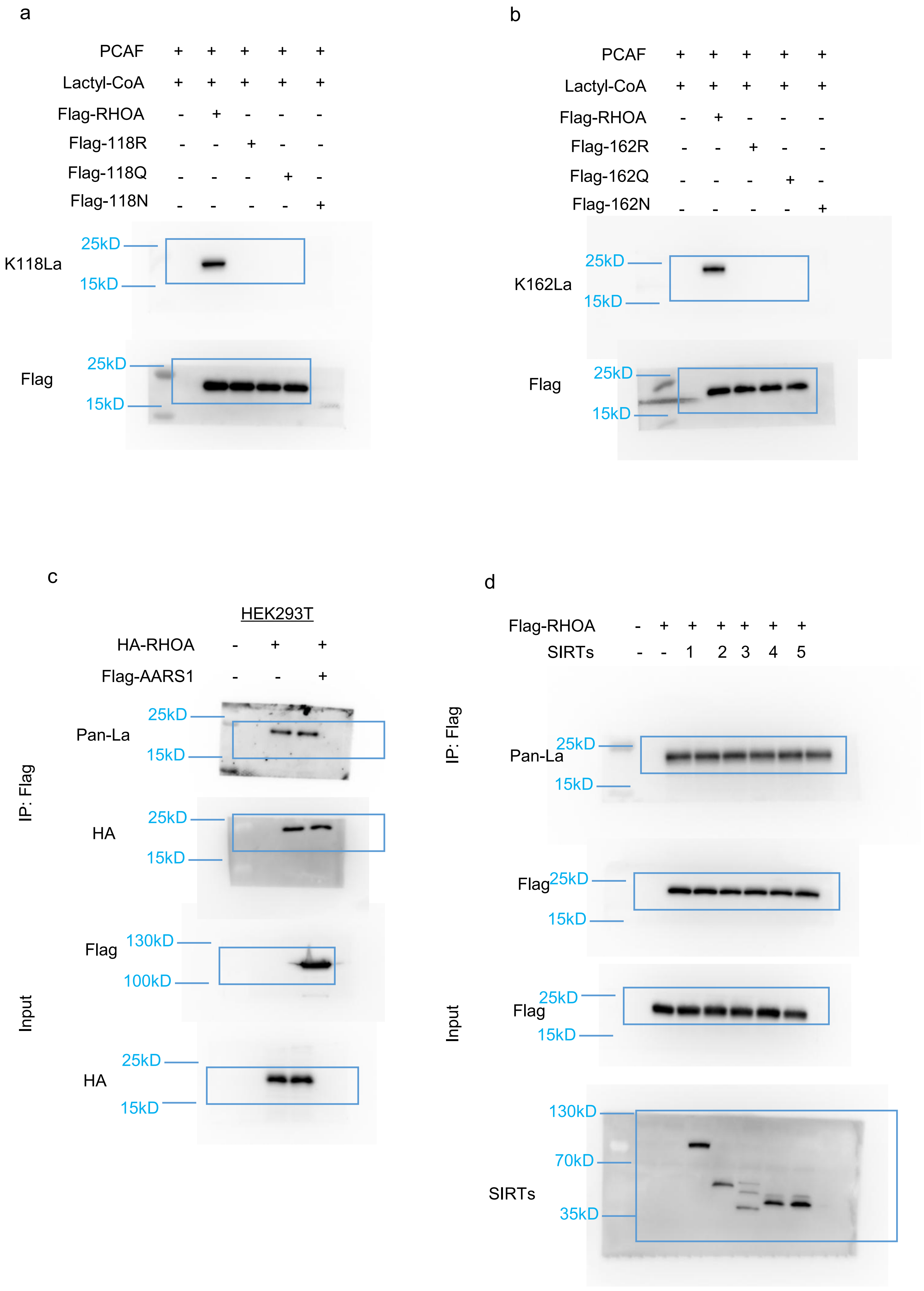

Supplementary Fig 7

e

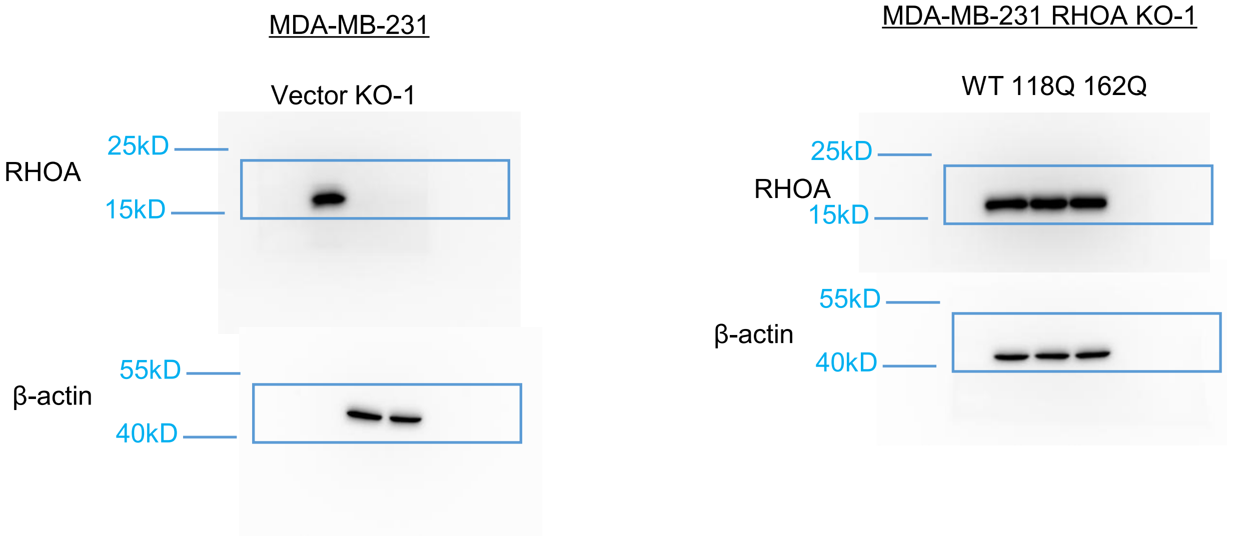

Supplement: Supplementary file 1 — Supplementary Material 1: Supplementary Fig. 1. a-c, Kaplan-Meier survival analysis for OS (a), RFS (b) and DMFS (c) of patients in an aggregate breast cancer dataset according to RHOA expression status. The p value was determined using the log-rank test. d, e, SUM159 cells were transfected with Flag-RHOA plasmid and cultured in different concentrations of glucose (d) or sodium lactate (e) for 24 hours, followed by immunoprecipitation. The Kla levels of precipitated protein were analyzed by Western blot. f, g, MDA-MB231 (f) and SUM159 cells (g) were cultured in different concentrations of sodium lactate for 24 hours, followed by immunoprecipitation. The Kla levels of precipitated RHOA were analyzed by Western blot. h, Amino acid sequence logo of conserved G4 and G5 motifs in the RAS superfamily. i, j, WT-RHOA, K118R or K162R mutants were expressed in SUM159 cells, and lactylation of K118 (i) and K162 (j) was determined following immunoprecipitation. k, Kla was selectively incorporated into RHOA-K118 or K162TAG-EGFP in HEK293T cells using the Mb-Pyl Kla-RS/Pyl-tRNA pair; green fluorescence (bottom) and bright field (top) images were obtained to confirm the expression of EGFP in the absence or presence of 2 mM Kla. l, Following Kla was selectively incorporated into Flag-RHOA-K118 or K162TAG in HEK293T cells, expression of lactylated RHOA was analyzed by Western blotting. Supplementary Fig. 2. a, b, ROCK2-RBD-HA was co-expressed with WT (WT), lactylated (a), or different mutant RHOA (b) in HEK293T cells, and the immunoprecipitated ROCK2-RBD-HA was examined by Western blotting. c, Purified GST and GST-RBD were detected by Coomassie blue staining. d, WT-RHOA or different mutants were expressed in HEK293T cells, and their activities were determined by GST-RBD pull-down assay. e, Expression of p-MLC2 and MLC2 in HEK293T with WT-RHOA or lactylated RHOA was examined by Western blotting. f, Stable knockdown of RHOA expression was established in MDA-MB231 and SUM159 cells. RHO [file 12943_2025_2511_MOESM1_ESM.zip › supplementary materials/the full uncropped Gels and blots images_RHOA_LA.pdf]
