# Supplementary material for: RHOA lactylation at oncogenic hotspots promotes oncogenic activity and protein stabilization
Source: Mol Cancer. 2025 Nov 25;24:311. doi: 10.1186/s12943-025-02511-7 (PMC12750741; doi:10.1186/s12943-025-02511-7)
Supplement: Supplementary file 1 — Supplementary Material 1: Supplementary Fig. 1. a-c, Kaplan-Meier survival analysis for OS (a), RFS (b) and DMFS (c) of patients in an aggregate breast cancer dataset according to RHOA expression status. The p value was determined using the log-rank test. d, e, SUM159 cells were transfected with Flag-RHOA plasmid and cultured in different concentrations of glucose (d) or sodium lactate (e) for 24 hours, followed by immunoprecipitation. The Kla levels of precipitated protein were analyzed by Western blot. f, g, MDA-MB231 (f) and SUM159 cells (g) were cultured in different concentrations of sodium lactate for 24 hours, followed by immunoprecipitation. The Kla levels of precipitated RHOA were analyzed by Western blot. h, Amino acid sequence logo of conserved G4 and G5 motifs in the RAS superfamily. i, j, WT-RHOA, K118R or K162R mutants were expressed in SUM159 cells, and lactylation of K118 (i) and K162 (j) was determined following immunoprecipitation. k, Kla was selectively incorporated into RHOA-K118 or K162TAG-EGFP in HEK293T cells using the Mb-Pyl Kla-RS/Pyl-tRNA pair; green fluorescence (bottom) and bright field (top) images were obtained to confirm the expression of EGFP in the absence or presence of 2 mM Kla. l, Following Kla was selectively incorporated into Flag-RHOA-K118 or K162TAG in HEK293T cells, expression of lactylated RHOA was analyzed by Western blotting. Supplementary Fig. 2. a, b, ROCK2-RBD-HA was co-expressed with WT (WT), lactylated (a), or different mutant RHOA (b) in HEK293T cells, and the immunoprecipitated ROCK2-RBD-HA was examined by Western blotting. c, Purified GST and GST-RBD were detected by Coomassie blue staining. d, WT-RHOA or different mutants were expressed in HEK293T cells, and their activities were determined by GST-RBD pull-down assay. e, Expression of p-MLC2 and MLC2 in HEK293T with WT-RHOA or lactylated RHOA was examined by Western blotting. f, Stable knockdown of RHOA expression was established in MDA-MB231 and SUM159 cells. RHO [file 12943_2025_2511_MOESM1_ESM.zip › supplementary materials/supplementary figures.doc]

**SUPPLEMENTARY FIGURE LEGENDS**

**Supplementary Fig. 1.**

**a-c,** Kaplan-Meier survival analysis for OS (a), RFS (b) and DMFS (c) of patients in an aggregate breast cancer dataset according to RHOA expression status. The p value was determined using the log-rank test.

**d, e,** SUM159 cells were transfected with Flag-RHOA plasmid and cultured in different concentrations of glucose (d) or sodium lactate (e) for 24 hours, followed by immunoprecipitation. The Kla levels of precipitated protein were analyzed by Western blot.

**f, g,** MDA-MB231 (f) and SUM159 cells (g) were cultured in different concentrations of sodium lactate for 24 hours, followed by immunoprecipitation. The Kla levels of precipitated RHOA were analyzed by Western blot.

**h,** Amino acid sequence logo of conserved G4 and G5 motifs in the RAS superfamily.

**i, j,** WT-RHOA, K118R or K162R mutants were expressed in SUM159 cells, and lactylation of K118 (i) and K162 (j) was determined following immunoprecipitation.

**k**, Kla was selectively incorporated into RHOA-K118 or K162TAG-EGFP in HEK293T cells using the Mb-Pyl Kla-RS/Pyl-tRNA pair; green fluorescence (bottom) and bright field (top) images were obtained to confirm the expression of EGFP in the absence or presence of 2 mM Kla.

**l,** Following Kla was selectively incorporated into Flag-RHOA-K118 or K162TAG in HEK293T cells, expression of lactylated RHOA was analyzed by Western blotting.

**Supplementary Fig. 2.**

**a, b,** ROCK2-RBD-HA was co-expressed with WT (WT), lactylated (a), or different mutant RHOA (b) in HEK293T cells, and the immunoprecipitated ROCK2-RBD-HA was examined by Western blotting.

**c,** Purified GST and GST-RBD were detected by Coomassie blue staining.

**d,** WT-RHOA or different mutants were expressed in HEK293T cells, and their activities were determined by GST-RBD pull-down assay.

**e,** Expression of p-MLC2 and MLC2 in HEK293T with WT-RHOA or lactylated RHOA was examined by Western blotting.

**f,** Stable knockdown of RHOA expression was established in MDA-MB231 and SUM159 cells. RHOA expression in these cells was analyzed by Western blotting. Actin was used as a loading control.

**g**, Relative fluorescence intensity of F-actin in shRHOA-expressing MDA-MB-231 cells with stable WT-RHOA or K118Q mutant expression was analyzed usign ImageJ.

**h,** F-actin was stained with Rhodamine Phalloidin (Red) in shRHOA-expressing SUM159 cells with stable WT-RHOA or K118Q mutant expression.

**i,** Structural illustration of lysine lactylation, lysine acetylation and glutamine.

**Supplementary Fig.3.**

**a,** Interactions of K118 within the G4 motif and K162 within the G5 motif with GTP-bound RHOA (PDB: 4XOI). Hydrogen bond interactions are shown as dashed lines.

**b,** The correlation of K118 and K162 with GTP in the RHOA is represented by the crystal structure (PDB: 1FTN).

**c,** Overlay of the WT-RHOA crystal structure (PDB: 1FTN; tan) and modeled WT-RHOA (sky blue). A magnified overlay of GDP and GTP structure was shown on the right.

**d,** Overlay of WT-RHOA (tan) with K118Q (sky blue), K118R (pink), and K118N mutants (light green), respectively after modeling. A magnified overlay of GTP structure was shown on the right.

**e-g,** Overlay and histogram for protein backbone Cα RMSF plots of MD trajectories between WT-RHOA and K118Q (e), K118R (f), or K118N mutant (g).

**h,** The binding activity between GTP and purified RHOA, RHOA mutant (K118Q), or RHOA-K118La (from site-specific glutarylation system) were determined by GTP pull-down assay.

**i,** Heatmap for hydrogen-bonding interactions of GTP with WT-RHOA, K118Q, K118R, or K118N mutant. Occupancy is defined as the ratio of the simulation time that a particular hydrogen bond is present.

**Supplementary Fig. 4.**

**a,** MS/MS spectra of representative RHOA peptides carrying K118 ubiquitination.

**b,** WT-RHOA was expressed in HEK293T cells for 48 h, and then the cells were treated with or without 10 μM MG132 for 6 h. Cell lysates were analyzed by Western blotting.

**c,** For CHX assay, shRHOA-expressing SUM159 cells with WT-RHOA or K162 mutant expression were treated with 25 μg/mL CHX for the indicated time; the expression level of RHOA was examined by Western blotting.

**d,** Flag-RHOA was co-transfected with HA-ubiquitin into HEK293T cells. Ubiquitination of immunoprecipitated RHOA was measured.

**e,** HEK293T cells were co-transfected with the indicated plasmids, ubiquitination of K118R mutant was measured by ubiquitination assay.

**f,** Box-plots indicate KCTD13 mRNA expression in different subtypes of breast cancer from two datasets (MEBTABRIC and GSE25066).

**g,** Analysis of GSE25066 dataset for the relationship between KCTD13 expression and chemotherapy sensitivity.

**h-j,** Kaplan-Meier survival analysis for RFS, and DMFS of patients in the aggregate breast cancer dataset and the GSE25066 dataset according to KCTD13 expression status. The p value was determined using the log-rank test.

**k,** The lysates of HEK293T cells with WT-RHOA, K162R mutant, or/and KCTD13 expression were subjected to immunoprecipitation with Flag-M2 agarose, and then their interactions were analyzed by Western blotting.

**Supplementary Fig. 5.**

**a,** Table presenting a portion of the RHOA-binding proteins identified through proximity labeling mass spectrometry (PL-MS).

**b,** Box-plots indicate USP9X expression in different histological grades of breast cancer from the MEBTABRIC dataset.

**c-e,** Kaplan-Meier survival analysis for OS, RFS, and DMFS of patients in the indicated datasets according to USP9X expression status. The p value was determined using the log-rank test.

**f,** HEK293T cells were co-transfected with the indicated plasmids, ubiquitination of RHOA was measured by ubiquitination assay.

**Supplementary Fig. 6**

**a, b,** *In vitro* lactylation assays were used to examine the effect of PCAF on the lactylation of wild-type RHOA-K118 (a) and RHOA-K162 (b), and their corresponding site mutants.

**c,** HEK293T cells were transfected with RHOA and/or AARS plasmids, and the lactylation level of RHOA was measured using a pan-Kla antibody.

**d,** HEK293T cells were co-expressed with RHOA, and indicated SIRT, and the lactylation level of RHOA was measured using a pan-Kla antibody.

**e,** Kaplan-Meier survival analysis of patients in the indicated datasets according to HDAC3 mRNA expression status. The p value was determined using the log-rank test.

**Supplementary Fig. 7.**

**a,** The formation of colonies from shRHOA-expressing MDA-MB231 and SUM159 cells with stable WT-RHOA, K118Q, or K162Q mutant was measured. Data were presented as a percentage of empty vector cell lines (mean ± SD in three separate experiments). *p< 0.01 by Student’s t-test.

**b,** shRHOA-expressing MDA-MB231 cells with stable WT-RHOA, K118Q or K162Q mutant expression were injected into nude mice via the tail vein. After 4 wks, the development of lung metastases was examined in paraffin-embedded sections stained with hematoxylin and eosin.

**c, d,** Ki67 was analyzed by immunohistochemistry in the indicated tumor tissues from nude mice (mean ± SD in three separate experiments).

**e,** RHOA was successfully knocked out in MDA-MB231 cells (left). WT-RHOA, K118Q, or K162Q mutants were stably transfected into the RHOA-knockout MDA-MB231 cells (right). Western blotting was used to analyze RHOA expression in these cells. Actin was used as a loading control.

**f,** RHOA-knockout MDA-MB231 cells with stable WT-RHOA, K118Q or K162Q mutant expression were injected into nude mice via the tail vein. After 4 wks, the development of lung metastases was monitored using bioluminescence imaging (left) and quantified by measuring photon flux (mean of 6 animals + SEM) (right). Three representative mice from each group were shown (left).

**g,** F-actin was stained with Rhodamine Phalloidin (Red) in shRHOA-expressing SUM159 cells with stable K118Q mutant expression following treatment with or without Y-27632 (10 μM).

**h,** Glucose level was analyzed in normal tissue, luminal, and basal-like tumors from Tang’s metabolomics dataset.

**i,** Analysis of Johansson’s dataset for the expression of RHOA and LDHA. The relative level of RHOA was plotted against that of LDHA.
